# Occurrence, transformation, bioaccumulation, risk and analysis of pharmaceutical and personal care products from wastewater: a review

**DOI:** 10.1007/s10311-022-01498-7

**Published:** 2022-08-17

**Authors:** Uttpal Anand, Bashir Adelodun, Carlo Cabreros, Pankaj Kumar, S. Suresh, Abhijit Dey, Florencio Ballesteros, Elza Bontempi

**Affiliations:** 1 Ben-Gurion University of the Negev, 84105 Beer-Sheva, Israel; 2 Department of Agricultural and Biosystems Engineering, University of Ilorin, PMB 1515, Ilorin, Nigeria; 3 Department of Agricultural Civil Engineering, Kyungpook National University, Daegu, Republic of Korea; 4 Environmental Engineering Program, National Graduate School of Engineering, University of the Philippines, 1101 Diliman, Quezon City, Philippines; 5 Agro-Ecology and Pollution Research Laboratory, Department of Zoology and Environmental Science, Gurukula Kangri (Deemed to Be University), Haridwar, Uttarakhand 249404 India; 6 Department of Chemical Engineering, Maulana Azad National Institute of Technology, Bhopal, Madhya Pradesh 462 003 India; 7 Department of Life Sciences, Presidency University, 86/1 College Street, Kolkata, West Bengal 700073 India; 8 INSTM and Chemistry for Technologies Laboratory, University of Brescia, Via Branze 38, 25123 Brescia, Italy; 9 Zuckerberg Institute for Water Research, Jacob Blaustein Institutes for Desert Research, Ben Gurion University of the Negev, Midreshet Ben Gurion, 8499000, Israel

**Keywords:** Pharmaceutical and personal care products (PPCPs), Active pharmaceutical ingredients, Wastewater treatment plants, Environmental pollution, Human health risk assessment, COVID-19

## Abstract

Almost all aspects of society from food security to disease control and prevention have benefited from pharmaceutical and personal care products, yet these products are a major source of contamination that ends up in wastewater and ecosystems. This issue has been sharply accentuated during the coronavirus disease pandemic 2019 (COVID-19) due to the higher use of disinfectants and other products. Here we review pharmaceutical and personal care products with focus on their occurrence in the environment, detection, risk, and removal.

**Supplementary Information:**

The online version contains supplementary material available at 10.1007/s10311-022-01498-7.

## Introduction

Pollutants in wastewater streams vary according to their sources, but they typically consist of organic and inorganic chemicals, nutrients, solid wastes, oxygen-demanding wastes, pathogenic microorganisms, and micropollutants among a few. When these pollutants are released into the environment, they negatively impact the ecosystem, public health, and the economy as a whole (Harrison et al. 2006; Meyer et al. 2019; WWAP 2017; Iyer et al. 2021). The micropollutants identified as “emerging contaminants” or “emerging pollutants” include a wide spectrum of pharmaceutical and personal care product (PPCP) compounds. They constitute a large group of pollutants that originate from sources closely related to anthropogenic activities such as cosmetics, therapeutic drugs, personal hygiene products, agricultural and industrial effluents, and hospital streams. (Sangion and Gramatica 2016). From these point and non-point sources, several PPCP compounds are inadvertently released into the environment unmonitored and unregulated. Some of these PPCP compounds are listed by the United States Environmental Protection Agency as priority pollutants (Daughton 2004; Hoenicke et al. 2007).

Owing to the great benefits derived from the use of PPCPs, particularly pharmaceutical products, in controlling the spread of human and veterinary diseases, unregulated sale and misuse have been observed and reported in previous studies (Miyazaki et al. 2020; Rees et al. 2021). Self-medication among the population to treat common illnesses is documented in both developed and developing countries. Its prevalence worldwide approximately ranges from 12.8% to as high as 81.5% (Kassie et al. 2018). In connection with population growth and their accessibility, PPCPs are easily introduced into the environment and the water cycle.

Van Boeckel et al. estimated the global consumption of antimicrobials was 63,151 tonnes in 2010 and is projected to increase by 67% in 2030 (Van Boeckel et al. 2015). Also, due to the increasing trend of urbanization and shift in lifestyle worldwide, the consumption pattern of pharmaceuticals has gradually changed alongside the prevailing lifestyle-related ailments such as cardiovascular diseases and diabetes (Mohapatra et al. 2016). The consumption trajectory and pattern of PPCPs are further changed by the ongoing COVID-19 pandemic. This change in PPCPs consumption poses a challenge in the assessment of their occurrence, distribution, and reactivity to the environment (Wang and Wang 2016).

The interest in pharmaceutical and personal care products-related studies has noticeably increased over the past couple of years (see Fig. S1, in the supporting information). Therefore, this work presents state-of-the-art knowledge with a particular focus on recent advancements in PPCPs detection and removal technologies, ecological risk, and their assessment during the ongoing pandemic, based on a restricted and suitable number of papers selected by the authors (see Fig. S2, for a clarification of the bibliography selection procedure). On the basis of the results of the literature searches and bibliography cluster analysis (see Fig. S3 in the supporting information) the contents of this review paper have been defined.

The study is important and timely for researchers, practitioners, and policy makers working in the domain of environmental pollution and health management including wastewater systems.

## Occurrence

PPCPs are detected in different environmental compartments, showing that they cannot be removed by conventional treatments (Wang et al. 2019; Wang and Chen 2020a). For instance, clofibric acid and salicylic acid are detected in river wastewater and sewage through detection techniques (Garrison et al. 1976; Hignite and Azarnoff 1977). Caffeine is present in domestic wastewater (Yang et al. 2013). Surface waters contain more than 50 pharmaceuticals detected in 139 streams across 30 states in the USA (Kolpin et al. 2002). PPCP are found in sewage treatment plants in the southern parts of India (Subedi et al. 2015). These authors detected amphetamine, saccharin, cyclamate, and sucralose with concentrations of 4300 ng/l, 303,000 ng/l, 3460 ng/l, and 1460 ng/l, respectively. From an average daily sewage flow rate of 20.7 million litres received from a population of 325,000, Subedi et al. estimated the daily discharge mass of amphetamine, and saccharin at 6.93 kg, and 2.52 kg, respectively (Subedi et al. 2015). It should be noted that the discharge concentration limit of pharmaceuticals in groundwater and surface water is less than 100 ng/l, and in the case of drinking water, less than 50 ng/l (WHO 2012).

The occurrence of PPCPs in river systems is widely studied mainly because rivers have a vital role in anthropogenic and socio-economic activities (Peng et al. 2017; Roberts et al. 2016; Sharma et al. 2019; Yang et al. 2013). PPCPs are discharged into river systems mainly from wastewater drains, effluents from wastewater treatment plants, and water runoffs during rainy periods (Kumar et al. 2019; Mutiyar and Mittal 2014; Prabhasankar et al. 2016; Scott et al. 2014).

**Fig. 1:**
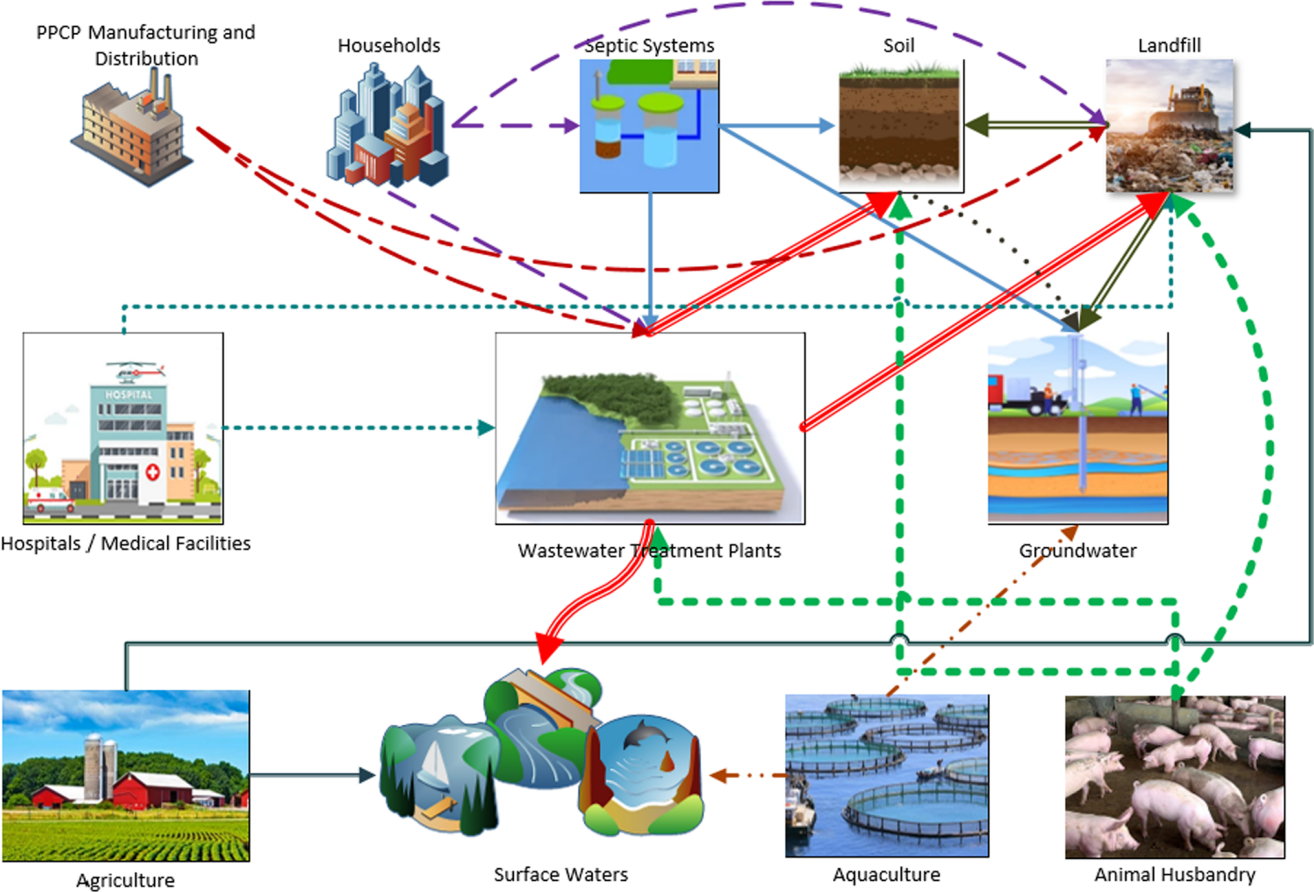
Sources and pathways of pharmaceuticals and personal care products (PPCP) in the environment. Flowchart depicting the routes of products originating from various sources such as industry, households, landfills, hospitals, agriculture, aquaculture, animal husbandry, and wastewater treatment facilities, which lead to the contamination of different environmental compartments including soil, surface water bodies, groundwater, and agricultural lands

In India, rivers are the main sources of drinking water and irrigation. And noting that India is ranked 13^th^ in terms of consumption of pharmaceutical products globally (Mutiyar et al. 2018), it’s therefore not a surprise that several studies about the occurrence of PPCPs in some of the major Indian rivers were conducted (Balakrishna et al. 2017), such as Yamuna (Mutiyar et al. 2018), Ganges (Sharma et al. 2019), and Brahmaputra (Kumar et al. 2019). The study conducted by Singh and Suthar mainly focused on caffeine, triclosan, acetaminophen, and tetracycline in the Ganges River (Singh and Suthar 2021). They detected an overall concentration of PPCP compounds in the range below the detectable limit to 1104.84 ng/l. Based on their study, the presence of PPCPs which tend to negatively impact both algae and fish biota was revealed. There are also PPCP studies that were done in conjunction with studies about pathogenic microorganisms, just like what Kumar et al. had done (Kumar et al. 2019). They performed identification of PPCPs and viruses in the Brahmaputra River. They were able to detect PPCP compounds such as acetaminophen, caffeine, theophylline, crotamiton and carbamazepine, and pathogenic microorganisms such as Aichi, pepper mild mottle, hepatitis A, norovirus GI, GII (Kumar et al. 2019). Figure 1 illustrates the diverse pathways with which PPCPs are introduced into the environment.

With the world population growth and relative improvement in global living conditions and lifestyles, the use of PPCPs has become very widespread and ubiquitous more than ever before. This is evident and prevalent across all socio-economic levels of the population. The pseudo-persistence of PPCP compounds in the environment is brought about by the routine, daily use of consumer products that contain the active PPCP compounds. The PPCP compounds are released into the environment regularly, albeit, at low or trace concentrations. This is mainly because PPCPs include a wide variety of products. From human prescription and non-prescription drugs, illicit drugs, veterinary drugs, hormones, and to consumer products such as fragrances, toothpaste, laundry detergents, and skincare (e.g., soap, sunscreen, lotion), haircare (e.g., shampoo, conditioner, gel), and disinfectants. With such a vast selection of products that are frequently used, PPCP compounds are easily released into the environment.

Starting from the bulk production of the active pharmaceutical ingredients, and the subsequent manufacturing, quality control and assurance and post-production processes of the medicinal and personal hygiene products, wastewater and solid wastes laden with PPCP compounds are already generated (Shalini et al. 2010). The wastewater generated during the manufacturing processes can either be treated using decentralized or localized or centralized wastewater treatment systems. A decentralized wastewater treatment system pertains to onsite treatment facilities, specifically put up and managed by the manufacturing plants or business enterprises themselves, to treat the wastewaters they generate (Singh et al. 2019). This is usually practised by manufacturing plants located far outside of the industrial zones and far away from urban areas and is not connected to the sewerage network coverage.

A centralized wastewater treatment system, on the other hand, refers to wastewater treatment facilities designed to service urban areas or industrial hubs with established sewerage network infrastructure. In terms of capacity, a centralized wastewater treatment system can treat a higher volume of wastewater. About the solid wastes originating from the manufacturing process of PPCPs, they are typically discarded into landfills and garbage dump sites or incinerated. Landfill waste disposal is commonly practised in developing countries, notwithstanding its negative health and environmental impacts, because it is the cheapest method to dispose of solid wastes that are generated (Bong et al. 2017; Anand et al. 2021a, b, c, d).

When pharmaceutical products are ingested, as much as 10–90% of the active pharmaceutical ingredients are excreted, unchanged, in their original compound form and some portions are transformed as metabolites (Zuccato et al. 2005). The excreted pharmaceutical compounds and metabolites make their way into wastewater treatment plants when flushed from toilets, which ultimately flow into the treatment plants. With that said, hospitals and medical facilities can be considered major sources of PPCP wastes considering that in-patients are treated there with medications to recuperate from illnesses they suffer. Some patients even have to stay for an extended period in hospitals before they can fully recover. The utilization of hospitals in some cities across the globe has even reached critical levels with the advent of the COVID-19 pandemic in the latter part of 2019 (Adelodun et al. 2021a, b, c, d; Anand et al. 2021a, b, c, d) and the succeeding waves of infection. Patients who suffer from severe symptoms of the disease need a longer time to convalesce.

Considering the absence of medicine specifically formulated to combat COVID-19 infection, several pharmaceutical interventions have been put into a clinical trial or implemented to symptomatically treat COVID-19 patients (Ibrahimagić et al. 2020; Anand et al. 2021a, b, c, d). With the looming paranoia about the health risk brought about by the pandemic, self-medication among the population has increased, in the hope of preventing COVID-19 infection or relieving symptoms, they experience (Malik et al. 2020). The presence of two among the approved medications prescribed to COVID-19 patients, Remdesivir, Dexamethasone, and their metabolites in surface waters have already been detected by (Desgens-Martin and Keller 2021). In addition, the pandemic has also prompted the frequent use of disinfectants (Dewey et al. 2021; Ghafoor et al. 2021), and also an increase in the use of antibiotics (Chen et al. 2021; Pérez de la Lastra et al. 2022).

Compared to pharmaceutical products, however, personal care products are more widely used daily, therefore the wastes, both solid wastes, and wastewaters, generated from their usage are greater in quantity and scope. The personal care products wastewaters are transported from the household sinks and drainage to the wastewater treatment plants. In contrast, households outside the sewerage system grids, are directly releasing the untreated wastewaters they generate to the environment through open canals that wind up to the nearest surface waters they flow to. They also contaminate the soil surfaces they come in contact with along the way, and over time, even the groundwater. It should be highlighted that approximately 80% of worldwide wastewaters are directly released to the environment, untreated (WWAP 2017). From this information, it can be inferred that a significant amount of personal care products compounds presents in wastewaters directly contaminate the natural environment they flow into, both soil and water surfaces.

Along with population growth, there has been an increase in demand for meat products. This gave rise to the practice of intensive livestock activities and farming to meet consumer demands. And to shorten the harvest time of meat from animal husbandry (e.g., poultry, hog, cattle) and aquaculture, animal feeds are supplemented with antibiotics, vitamins, and growth promoters. Antibiotics are added to prevent diseases from spreading among animals raised in closed quarters or limited spaces, and with high population density. Like human pharmaceuticals, veterinary pharmaceuticals are also excreted by animals in their original compound forms and as metabolites. However, unlike human excreta, animal excreta could be directly released into the environment in the case of animals grown in pastures or for setups where the domesticated animals are free-roaming. Veterinary pharmaceuticals and metabolites in animal manure are indirectly spread to agricultural lands when they are used as fertilizer to improve the conditions of the soil for planting. And in episodes when surface runoff occurs, the water contaminated with veterinary pharmaceuticals ends up in nearby water bodies, and overtime leaches to the groundwater sources.

Aquaculture done in open waters, such as lakes, is a direct source of veterinary pharmaceuticals and metabolites in the environment. The fish feeds fortified with antibiotics or growth promoters directly come in contact with the water body, thus releasing the pharmaceutical residues into the water ecosystem.

Since PPCPs have an expiry, the expired and unused products from households, in most cases, either end up flushed in toilets or sinks, or thrown out along with other household solid wastes and get delivered into landfills. It should be noted that expired PPCPs would still have a significant amount of active pharmaceutical ingredients. Some products can maintain above 90% of the claimed amount of active pharmaceutical ingredients way past their expiration (Mani and Thawani 2019). Therefore, the proper handling of expired PPCP is very important in controlling PPCPs in the environment. However, the disposal practice of PPCPs stems from the risk perception of the population in general (Binti Muhamad and Binti Mohamed Zuki 2020). The lower the awareness of an individual about the risk of PPCPs to the environment, the more likely the individual will dispose of expired PPCPs carelessly. Although there may be mechanisms to recover the unused and expired PPCPs, the facilities to make it possible are limited, most particularly in developing countries. As much as 29% of expired PPCPs are released into the environment, untreated (Esseku 2016).

Landfill, being mainly used as means to dispose of municipal solid wastes, becomes a critical point where PPCP contamination of groundwater tables can occur. The leachate from the landfill can seep through the soil layer and eventually the groundwater over an extended period.

### Wastewater treatment plants as accumulation points of pharmaceuticals and personal care compounds

Generally, in urban area settings, the infrastructure for the sewerage system is more defined and developed (Wang et al. 2020). Therefore, the sewage effluents from households and businesses are gathered together and transported through the sewerage system to the wastewater treatment plants. This makes wastewater treatment plants a hotspot for micropollutants derived from PPCP usage because they gather them together in one location. Even at present time, the commonly used wastewater treatment methods are not able to completely remove PPCPs because they are not designed specifically to neutralize or remove such contaminants from the wastewater stream. In addition, the regulatory effluent standards, particularly in developing countries, do not include PPCP concentrations in the regulated parameters, thus, they are inadvertently released into the environment, uncontrolled and unmonitored. Several studies have reported the presence of PPCPs in water bodies where effluents from wastewater treatment plants are released (Burns et al. 2018; Kanama et al. 2018; Mohapatra and Kirpalani 2019). Table S4 (see Supporting information) provides an overview of some of the PPCP compounds that have been detected from the effluents of wastewater treatment plants and the adjacent receiving bodies of water Ashfaq et al. (2017) Franklin et al. (2016) Lin et al. (2018) Nazari and Suja (2016) Williams et al. (2003). Among the PPCP compounds listed, acetaminophen, caffeine, carbamazepine, diclofenac, ibuprofen, and sulfamethoxazole are frequently detected.

Apart from the effluents from wastewater treatment plants, the biosolids produced from activated sludge treatment processes also contain PPCP compounds. They contaminate agricultural lands when they are applied as fertilizers or as soil conditioners, just like animal manures.

### Transformation products

Drug metabolism is the metabolic breakdown or biotransformation of pharmaceutical substances in living organisms, usually through enzymatic actions. Drugs can be biotransformed through oxidation, reduction, hydrolysis, hydration, conjugation, condensation, or isomerization. The majority of the metabolic processes involving pharmaceutical substances occur in the liver, but some also occur in the epithelial cells of the upper portion of the intestines, lungs, kidney, placenta, and even the brain (Bachmann 2009). Drug metabolism occurs in the said organs because the enzymes that enable the reactions are located there. Drugs administered through intravenous infusions have biotransformations that occur mainly in the liver. In contrast, ingested pharmaceutical substances undergo biotransformation both in the intestines and liver (Bachmann 2009; Stanley 2017). For topical medicines, their effectivity is directly related to their ability to be absorbed through the skin. The fraction of the active pharmaceutical ingredients remains on the skin surface and can easily be washed and rubbed off. The liver, and subsequently the kidney, play essential roles in the removal of pharmaceutical compounds from the blood stream, and ultimately from the body through excretion.

As regards to PCPs or hygiene products, the majority of these are externally applied, i.e., on hair and skin. Therefore, they are easily removed when individuals who use them wash, take a bath or dip in pools or on beaches. Externally applied PPCPs can also be rubbed off to clothing, objects, and surfaces that individual come in contact with.

The excreted and washed-off PPCP compounds and pharmacologically active metabolites undergo further degradation in the environment as they come in contact with sunlight, air, water, soil, microorganisms, and other physical entities or forces. PPCP compounds have relatively shorter half-lives in the environment as compared to persistent organic pollutants (Yin et al. 2017). And knowing this, it can be inferred that PPCP compounds are more likely to form transformation products in environment matrices as they degrade. Some PPCP compounds are known to be light-sensitive, meaning, they easily degrade and transform when exposed to sunlight or ultraviolet light. Some PPCPs are easily oxidized and transformed by mere contact with air. Because of this, some studies and research have focused on the photolysis (Kim and Tanaka 2009; Luo et al. 2018) and oxidation (Wang et al. 2015) of PPCP compounds as viable treatment methods to remove these pollutants from wastewater streams. However, the focus mostly in this type of study is on the parent PPCP compounds, and only a little attention is given to the degradation byproducts or transformation products. This is because the analysis of PPCPs in different environment matrices is very laborious and costly. Analyses are usually conducted for targeted compounds rather than the identification of all substances in a given sample. There are thousands of PPCP compounds that are in use, but only a fraction of these substances has been studied in environmental compartments so far, thus, the knowledge gap about the comprehensive identification of PPCPs in the environment is enormous. There is even a bigger knowledge gap when it comes to the transformation products of the PPCP compounds.

To study the possible transformation products of PPCP compounds in the environment, experiments had been conducted in laboratory setups to determine the transformation products of specific compounds. It was shown that the generation mechanism of free radicals and the degradation mechanism of pollutants are not yet clear (Wang and Zhuan 2020) and that the toxicity of wastewater can change during the treatment (Wang and Wang 2021). Among the PPCP compounds that are frequently detected in the water environment, diclofenac and carbamazepine are the two of the most commonly reported contaminants in the past 10 years (Wang and Wang 2017; Wang et al. 2018a, b; Zhang et al. 2020). Carbamazepine has been determined to be recalcitrant to conventional wastewater treatment methods. Pan et al. investigated the degradation of carbamazepine by chlorine under ultraviolet irradiation and were able to identify 24 transformation products (Pan et al. 2017). On the other hand, diclofenac was found to have 13 phototransformation products based on a photolysis experiment done in water under direct solar irradiation (Agüera et al. 2005). This means that diclofenac can at least have 13 transformation products purely from exposure to sunlight when it is released to surface waters. PPCPs transform the environment via physical, chemical, and biological processes, so their transformation products can be more than what is currently known.

Apart from the parent PPCP compounds, their transformation products can also exhibit pseudo-persistence in environmental matrices. Some transformation products can be more persistent or more dangerous than the parent compounds (Cory et al. 2019; Kosjek and Heath 2008). For example, two of the phototransformation products of Naproxen were found to be more toxic than the parent compound (Cory et al. 2019). Based on information gathered from laboratory studies, transformation products formed during advanced oxidation processes can also be more toxic than the parent PPCP compounds (Yin et al. 2017). The current scope of their impact on the environment could not be measured mainly due to the huge knowledge gap regarding the occurrence of transformation products in environmental compartments.

## Persistence, bioaccumulation and health risk assessment

Before the 1960s, PPCP compounds in the aquatic environment, runoff streams, marine waterways, groundwater, and drinking water were undetected even though medicines were already widely used for human and veterinary purposes. This was due to limitations in analytical methods and technology available at that time, which were not designed to detect and identify compounds in environmental matrices at trace levels. Since 1960, consumption of pharmaceuticals has increased annually around the world (Ortiz de García et al. 2013), so the amount of PPCP compounds that are inadvertently released into the environment also increased.

The risks that PPCP compounds can pose include direct and indirect effects such as the impact on biochemical processes, disruption of the endocrine system, development of antimicrobial resistance, and the bio-accumulation of pharmaceuticals in non-target organisms (Frédéric and Yves 2014; Vasquez et al. 2014). Collado et al. reported the accumulation of active and inactive metabolites in the aquatic environment because of improper disposal of PPCP compounds (Collado et al. 2014). These PPCP compounds in surface waters may enter the food chain when non-target organisms bioaccumulate them (e.g., aquatic and riparian biota) (Richmond et al. 2018). Further, human-related pharmaceuticals in surface waters also affect the natural detoxification capability in fish populations by negatively impacting their metabolism processes (Burkina et al. 2015) Yeh et al. (2017) and diversity (Kuzmanović et al. (2016)).

Many studies and reports have confirmed that several sources including discharge effluents from industrial activities and hospitals, leaching from domestic septic tanks, runoff stream from farms, and improper disposal of PPCPs are significantly contributing to environmental pollution (Fenech et al. 2013; Iglesias et al. 2014; OECD 2019; WHO 2012). Practically, there are two ways with which PPCPs (human pharmaceuticals specifically) are released to the environment; (1) manufacturing faults, and disposal of unused (or expired) drugs into sinks and toilets or waste bins, which end up in landfills or incineration facilities; (2) excretion, and effluents from inefficient wastewater treatment plants (Vellinga et al. 2014). In landfills, the concentration of PPCP compounds that accumulate in leachates can be similar to or higher than the influent concentration of PPCPs in treatment plants (BIO Intelligence Service 2013; Clarke et al. 2015).

Excreta from humans, who have ailments and are under medications, contain PPCP compounds which end up in sewage streams (Li et al. 2019). However, conventional wastewater treatment methods are unable to completely remove these PPCP compounds and are instead released into receiving water bodies (Rodriguez-Narvaez et al. 2017). Take for example the removal efficiency of PPCP compounds such as carbamazepine and ibuprofen in wastewater treatment plants which was found to be at 8.1% and 87.5%, respectively (Santos et al. 2009). This means that about 91.9% and 12.5% of carbamazepine and ibuprofen concentration in the influent stream of wastewater treatment plants are discharged to the environment. Further, it can be seen that carbamazepine is more recalcitrant to conventional treatment processes than ibuprofen. The recalcitrance of PPCP compounds, along with other pollutants, from conventional wastewater treatment processes, has been the focus of research in recent years (Adelodun et al. 2019; Krzeminski et al. 2019). The incomplete removal of pollutants during treatments leads to their dispersion in water and soil matrices (Adelodun et al. 2019; Medrano-Rodríguez et al. 2020). Although PPCPs are usually detected at trace concentrations in environmental compartments, however, long-term exposure to these compounds can cause risk to human health and non-target organisms, that’s why there is a need to develop low-cost removal technologies to eliminate PPCP compounds from waste streams (Rodríguez-Narvaez et al. 2020).

Due to the pseudo-persistent characteristics of PPCPs in surface waters that receive effluents discharged from wastewater treatment plants, non-target organisms such as planktons (Yang et al. 2020), molluscs (de Solla et al. 2016), and fishes (Arnnok et al. 2017; Chen et al. 2017; Muir et al. 2017), had been documented to bioaccumulate them. This has serious consequences mainly because through bioaccumulation, PPCP compounds enter the food chain, and thus could pose risk to human health. Apart from surface waters, agricultural lands, where biosolids from wastewater treatment plants are used as conditioners, also becomes an entryway of PPCPs to the food chain through plant uptake of the residual PPCP compounds present in the biosolids (Keerthanan et al. 2021).

The persistence of PPCPs in surface waters such as rivers and lakes, where drinking waters are sourced, also becomes a window through which they could pose risk to the health of communities (Yang et al. 2017). Meprobamate, which is used to treat anxiety disorders, had been detected (40 ng/l) in the drinking water (Benotti et al. 2009). Both phenazone and propylphenazone were also found in drinking water (Reddersen et al. 2002; Zühlke et al. 2004). Another study reported the detection of antibiotics, beta blockers and antiepileptic drugs (below 100 ng/l) in the drinking water in the Netherlands (Mons et al. 2003).

The ongoing COVID-19 pandemic has spurred an inter-disciplinary and technological approach as a roadmap for water and wastewater management to help fight COVID-19, and possible future pandemics (Adelodun et al. 2021a, b, c, d; Anand et al. 2021a, b, c, d; Kareem et al. 2021; Tiamiyu et al. 2021; Anand et al. 2022). A similar approach can be adopted for studies regarding PPCPs and their characteristic behaviours such as persistence, bioaccumulation and toxicity.

The pandemic has brought about the increased usage of PPCPs such as antibiotics and disinfectants, which could pose consequent risks to the environment and non-target organisms or wildlife. Drugs used as therapeutic interventions for COVID-19 infection such as hydroxychloroquine, tocilizumab, sarilumab, and ritonavir have a two-fold increase in their usage (Aitken 2020). Also, the occurrence of the SARS-CoV-2 virus, which causes the COVID-19 disease, in wastewater streams poses a great challenge to wastewater treatment management. Bandala et al. had done a critical review on this aspect along with relevant associated technologies that could help address the issue (Bandala et al. 2021).

The risk potential of drugs being used as therapeutic interventions for the COVID-19 disease cannot be discounted as they will be continuously released to wastewater streams for as long as the threat of the disease exists. In addition to the pseudo-persistent PPCPs in environmental compartments like caffeine, diclofenac, carbamazepine, and others, the introduction of COVID-19-related drugs into the mix will make the multifaceted problem more complex. Their long-term effect on aquatic systems and human health is worth looking into as PPCP compounds can be more toxic, persistent, and mobile in the environment when compared to other chemical compounds (Bandala and Rodriguez-Narvaez 2019). One challenge that needs to be overcome, however, is the limited availability of information about the mass balance for COVID-19-related drugs and their metabolites being released into sewage streams.

The World Health Organization, in a report, has assessed that sectors like the pharmaceutical industry struggle to maintain natural market flow during pandemics, which leads to inaccessibility of essential medicines at affordable prices (WHO 2003). Disruption in the supply chain is felt by countries that are heavily dependent on the importation of active pharmaceutical ingredients. Take for example Iran, which imports 50% of its active pharmaceutical ingredient requirements (Cheraghali 2017) was impacted during the current pandemic. Ayati et al. Described an impressive COVID-19 impact on the pharmaceutical market and suggested evidence-based planning to overcome the challenges (Ayati et al. 2020). Many regulatory authorities have confirmed a shortage of prescribed medicines for hospitalized patients suffering from COVID-19 infection. For example, chloroquine and hydroxychloroquine, azithromycin, albuterol metered-dose inhalers, and some other sedation medications were listed to be in shortage in the USA due to their high demand in association with COVID-19 treatment (Bookwalter 2021). Also, some of the countries which import non-COVID-19 related drugs, such as pain relievers, had experienced delays due to the delivery priority of urgently needed medicines. These disruptions can lead to a slow-down of industry growth, supply chain, and long-term impact on the health and pharmaceutical market.

Before the pandemic, the European Union (European Commission 2019) had drawn new guidelines for the foreign investors, especially for the health market, and stated that export must be subjected to evaluation of risk assessment with the fulfilment of its citizen’s medicines requirement. The national drug policy will be revised according to the situation, and its policy shall be updated from time to time regarding components in the pharmaceutical sector such as price control, overstock, generic-based medicine, import of pharmaceutical ingredients, etc.

Therefore, governments’ assistance to the pharmaceutical industry is needed to address concerns or issues related to COVID-19, and governments should encourage research and development activities regarding balanced treatment strategies with optimistic medicines supply chain.

Due to COVID-19, the antiviral drugs used as therapeutic interventions for the disease have a high probability to be released into the environment. And when animals that are natural reservoirs of viruses are exposed to these PPCPs may induce antiviral selective pressures and viral mutations which can lead to antiviral drug resistance (Kumar et al. 2020). It should be noted that the SARS-CoV-2 virus is suspected to have originated from animal source (Andersen et al. 2020). Therefore, risk assessment of COVID-19-related pharmaceuticals is essential to prevent consequential negative impacts on human health.

In general, acceptable daily intake statistical calculation is established for assessing chemical risk in food and drinking water. The acceptable daily intake calculation is based on extrapolation factors that involve uncertainty and can be applied to a selected point of departure which is set from the epidemiological and toxicological database (FAO/WHO 2009). Point of departure can be also ensigned from two chemical additional factors of uncertainty, the concentration at no adverse effects, called as no-observed-adverse-effect level and the concentration at the lowest called as lowest-observed-adverse-effect level. There are few scientific reports available in the literature for health risk assessment of pharmaceuticals, especially about the lowest-observed-adverse-effect level factor in drinking water.

The minimum therapeutic dose is usually assessed for health risks in pharmaceuticals containing water. This minimum therapeutic dose is used for developing conservative screening values in point of departure (WHO 2012). World Health Organisation gave guidelines and protocol for developing screening values of chemicals in drinking-water quality. These values are useful to support decision-making criteria in the design of treatment plant (WHO 2012).

## Detection, determination, and extraction methods

The increasing use of pharmaceutical and personal care products which are regarded as emerging micropollutants or trace organic compounds in the environmental compartments has raised serious concerns about their potential ecological and health risks due to their recalcitrant, ubiquitous, and bioaccumulative nature (Dai et al. 2014; Ebele et al. 2017; Zhang et al. 2021). According to the United States Food and Drug Administration, over 20,000 prescription drug products were approved for marketing as of the year 2020, while there were about 1600 animal drug products (US FDA 2020). Non-prescription drugs accounted for about 51% of the specific classes of the PPCPs, with compounds like fragrances being underrepresented in the available literature (Meyer et al. 2019).

Moreover, the emergence of COVID-19 has led to excessive production and use of medications and health care products including disinfection by-products and other PPCPs to treat the infected patients and to also prevent the spread of the virus (Adelodun et al. 2020a, b; Lin et al. 2020; Zaidi and Hasan 2021). It has also been reported that about 4000 different pharmaceutical compounds entered environmental compartments in Europe (Mompelat et al. 2009). However, the detection of these emerging contaminants from various environmental compartments and their further retrieval, especially via the conventional wastewater treatment plants have always been a challenging task due to their low concentrations, typically in the range of microgram/l to nanograms/l (Adelodun et al. 2021a, b, c, d; Marasco Júnior et al. 2019; Snyder et al. 2007; Zhang et al. 2021). Even though they appear at very low concentrations in environmental compartments or reclaimed wastewaters, the physicochemical properties and toxicological effects of the compounds of PPCPs are found to have negative effects on the biotic environment, including the development, growth, and reproduction of biota (Ajibade et al. 2021a, b; Cheng et al. 2021; Zhang et al. 2021).

Cheng et al. reported that sulfamethoxazole exhibits high ecological risk as indicated by the low predicted no-effect concentration value of 27 ng/l after treatment in a constructed wetland (Cheng et al. 2021). The municipal wastewaters containing these emerging pollutants often discharge them into the environment without adequate treatment, thus increasing the potential contamination risk with organic and chemical pollutants (Adelodun et al. 2020a, b). Various studies have also shown that wastewater treatment plants could partially remove the compounds of PPCPs (Nguyen et al. 2021; Petrie et al. 2015; Rosal et al. 2010), thereby serving as point source discharges of the various PPCPs in the environment (Dai et al. 2014).

The detection and measurement of the compounds of PPCPs in the environmental compartments are essential steps toward their retrievals and decontamination to prevent any potential ecological and health risks. However, some of the metabolites of the registered PPCPs present in the environment are inadequately documented due to the limiting factor of the analytical tool, thereby leading to their possible underestimation in different environmental media (Poynton and Robinson 2018). Despite this, there have been significant efforts in the development of different detection and measurement methods and techniques for the micropollutants or trace organic compounds of PPCPs in various environmental media. Reyes et al. reported 580 unique compounds of PPCPs from a total of 133 studies that investigated the occurrence of PPCPs from real samples, with 23 frequently occurring compounds, including carbamazepine, caffeine, diclofenac, ibuprofen, acetaminophen, sulfamethoxazole, triclosan, *N*,*N*-Diethyl-meta-toluamide, naproxen, clarithromycin, triclocarban, propranolol, bisphenol, bezafibrate, methylparaben, salicylic acid, ofloxacin, metformin, tramadol, atorvastatin, diphenhydramine, sertraline, and diltiazem in identified nine different media (Reyes et al. 2021).

Similarly, Petrie et al. (2015) reported the presence of about 70 pharmaceuticals including a total of 15 illicit drugs and stimulants in UK wastewaters, with some of which have concentrations range of 17–5790 ng/l at the final effluents or surface waters (Kasprzyk-Hordern et al. 2009), depending on their usages. The non-steroidal anti-inflammatory drugs, anti-depressants, ꞵ-blockers, antimicrobials, antiepileptic carbamazepine, sunscreen agents, and preservatives are regarded as the most studied PPCPs considering that they are highly prescribed and consumed (Petrie et al. 2015); as such become ubiquitous in the wastewater plants. The presence of PPCPs has also been reported in the biosolids (treated sludge), which are often generated during the anaerobic digestion of wastewater in the treatment plants due to their high level of persistence (Cortés et al. 2013). While some PPCPs were found to be at very low concentrations in the treated sludge, the concentration of bisphenol A, triclocarban, triclosan, ciprofloxacin, norfloxacin, and ofloxacin were reported to be above an average of 1 mg/kg in various studies (Gottschall et al. 2012; Guerra et al. 2014; Heidler et al. 2006; Lindberg et al. 2005; Lozano et al. 2012; Sabourin et al. 2012).

Meanwhile, the PPCPs exhibit different physicochemical properties with varying fates and transport in the soil matrix. For instance, such as triclosan and triclocarban have higher hydrophobicity of log *K*_*ow*_ 4.2–4.8, making them be retained within the soil matrix, while antibiotics like ciprofloxacin, norfloxacin, and ofloxacin, on the other hand, with a relatively wide range of mobility in the soil and high water-soluble (≥ 3.0 × 10^4^ mg/l), are likely to be found around surface waters (Morais et al. 2013; Petrie et al. 2015).

The use of analytical technology tools like mass spectrometers including orbitrap, quadruple with linear ion-trap, and quadruple with time-of-flight could detect some low concentrations of the pollutants in the environmental samples (Reyes et al. 2021; Rosal et al. 2010). Recently, Saka (2020) reviewed different chloroquine quantitative determination and detecting techniques including chromatography, electroanalytical, electrophoresis, ELISA (Saka 2020). High-performance liquid chromatography is one of the most frequently used detection techniques (Saka 2020) and electroanalytical methods are highly significant mainly in situ analysis of chloroquine and other PPCPs in effluent and surface water. In a recently advanced carbon-graphene-based sensor, Lorenzetti et al. detected tetracycline in very concentration using reduced graphene oxide (Lorenzetti et al. 2020), Setznagl and Cesarino detected low concentrations of estriol hormone and glyphosate in water sample using reduced graphene oxide–metal nanoparticle (Setznagl and Cesarino 2020). Among all other metallic nanoparticles, the copper-based nanoparticle is best to detect PPCPs in effluent and surface water. However, sensor-based detectors are disposable after use, and some nanoparticles release into the environment which implies, drawbacks which is to be avoided through research and development of re-usable sensors and non-conventional nanoparticles. Costa-Rama et al. developed re-usable sensors for the detection of PPCPs in water (Costa-Rama et al. 2020). Xiang et al. reported PPCPs compounds detected in surface water or sediment in China (Xiang et al. 2021). They found that the concentration of caffeine, oxytetracycline, and erythromycin was higher in surface water. They found that 14 kinds of PPCPs compounds pose no significant risk through risk quotient criteria or assessment.

There are several advanced techniques and instrumentation for the detection of PPCPs compounds at very low concentrations which include gas chromatography with tandem mass spectrometry and liquid chromatography with tandem mass spectrometry (Ramos et al. 2019; Li et al. 2018; Rice and Mitra 2007; Trujillo-Rodríguez et al. 2018; Vega-Morales et al. 2010). The target compounds are depending on the type of method and physicochemical properties of particular chromatography (Lei et al. 2018; Huerta et al. 2013; Caldas et al. 2016; Arismendi et al. 2019). If target compounds are more soluble and polar in nature, liquid chromatography with tandem mass spectrometry analysis is the best choice (Meng et al. 2021). If the target compounds are more volatile, gas chromatography with tandem mass spectrometry analysis is a better choice (Fenech et al. 2013). Table S5 (in supporting information) reports the main instrumental techniques used for the detection of specific chemicals.

The major challenges of measurement of the compounds of PPCPs lie in the limit of detection of these emerging micropollutants, especially in the effluent samples (Bratkowska et al. 2011; Gilart et al. 2013; Kotnik et al. 2014) after treatment, due to improper sampling or calculation errors such as hydraulic retention time, leading to improper report or estimate of percentage removal (Rodriguez-Rodriguez et al. 2011; Snyder et al. 2007; Basaglia and Pietrogrande 2012). Ortega and co-workers found a wide range of uncertainty associated with the sampling methods which are dependent on the sampling site, a specific compound of interest, and the accuracy level of the analytical method employed (Ort et al. 2010a, b). Moreover, the majority of the studies reported having used existing traditional sampling methods, with only a few studies considered internationally acceptable guidelines when it comes to sampling and monitoring of PPCPs in environmental samples, especially when it is considered that sampling frequency could responsible for the concentration variations of the micropollutants (Ort et al. 2010a, b).

However, accurate presentation of PPCPs in environmental samples is not only dependent on sophisticated analysis but also on appropriate sampling methods. Unlike pharmaceuticals, the personal care products such as cosmetics and other additives that are used and applied externally are easily detected in the wastewater through which they are discharged via washing and bathing (Vallecillos et al. 2013; Santana-Viera et al. 2017; Kim et al. 2010). The selected compounds of PPCPs and their classifications along with their concentration in various environmental samples are presented in Table S6 (see supporting information) Batt et al. (2007) Bayati et al. (2021) Hamscher et al. (2002) Matongo et al. (2015) Mutiyar and Mittal (2013) Verlicchi et al. (2012). Decontamination and treatment methods (discussed in “Risk and ecotoxicological assessments, bioremediation, treatment technology, and removal methods” section) are also reported.

## Risk and ecotoxicological assessments, bioremediation, treatment technology, and removal methods

The toxicity of various micropollutants of PPCPs has continued to increase thus driving the need for awareness of their proper assessment in environmental media. For instance, diclofenac, a common anti-inflammatory pharmaceutical in environmental samples is considered to cause chronic and acute toxicity impacts on the various organs including the liver, and kidneys of living organisms (Vieno and Sillanpää 2014). Similarly, Petrie et al. analysed the toxicity of the most well-studied emerging contaminants of PPCPs based on the available data in the literature and classified them into harmful, toxic, and very toxic concerning their concentrations of 10 and 100 mg/l, 1 and 10 mg/l, and less than 1 mg/l, respectively (Petrie et al. 2015). The non-steroidal anti-inflammatory drugs such as diclofenac, acetaminophen, ibuprofen, naproxen, carbamazepine, trimethoprim, and lipid regulators including bezafibrate and clofibric acid-metabolite were classified as harmful contaminants, to aquatic organisms, ofloxacin, sulfamethoxazole, erythromycin, and oxytetracycline fall under toxic contaminants, while those with extremely low concentration had no adequate information to establish their impact on the environment and biota. Long-term exposure to acetaminophen, one of the most consumed PPCPs globally has been considered to cause cancer, endocrine disruption, and other several chronic diseases (Phong Vo et al. 2019). Also, among the micropollutants that are common to the United States, European Union, and China, antiretroviral Efavirenz and octocrylene were found to have the highest aquatic HazPi value, an index for measuring the persistence, bioaccumulation, bioactivity, and toxicity of emerging micropollutants (Fang et al. 2019).

Meanwhile, due to the various disinfectant byproducts formation from the continuous use of chlorine and ethanol-based disinfectants, coupled with some new pharmaceutical products for the treatment of COVID-19 infection and the detergent product for handwashing in preventing the spread (Adelodun et al. 2020a, b). There have been different technologies and techniques for the treatment of wastewater containing PPCPs, especially via wastewater treatment plants, which include sand filtration, sorption (adsorption and absorption), coagulation, ultrafiltration, advanced oxidation processes, ozone and ultraviolet light photolysis, bioremediation, and chlorine disinfection (see Table S6) Awfa et al. (2019) He et al. (2016). The removal efficiency of various PPCPs, however, is dependent on the appropriateness of the technology implemented in the wastewater treatment plants and other various factors which include system configuration, operation and treatment conditions, and influent loadings, making it difficult to compare removals of micropollutants in different treatment plants (Nam et al. 2014; Phong Vo et al. 2019). Nam et al. also reported the influence of seasonality in the concentrations of micropollutants in the influent of treatment plants, with metoprolol, one of the highly used beta-blockers, exhibiting recalcitrant and persistency during the treatment process (removal efficiency of 6%) (Nam et al. 2014). The primary treatment processes in treatment plants such as coagulation and sedimentation have been found inefficient in the removal of emerging micropollutants of PPCPs (Adams et al. 2002; Stackelberg et al. 2007; Vieno et al. 2007).

Thus, some studies suggested the use of alternative treatment methods or multiple treatment techniques that could be combined with the wastewater treatment plant for safe discharge of the effluent devoid of toxic emerging pollutants into the environment or the reuse of treated wastewater, especially for irrigation in agriculture, urban greening, and landscape and recreation (Carvalho et al. 2013; Lin et al. 2020; Nam et al. 2014; Rodriguez-Rodriguez et al. 2011; Vymazal et al. 2017). The combined treatment processes of coagulation-sedimentation, sand filtration, and disinfection were found to significantly remove some selected micropollutants, including carbamazepine, acetaminophen, and diclofenac in a wastewater treatment plant in Korea (Nam et al. 2014). New sustainable adsorbent materials have been also recently proposed for this aim (Bontempi et al. 2021; Fahimi et al. 2020).

The treated effluents (secondary) could either be reused for various purposes such as in industrial processes, in agricultural use for irrigation, in the urban landscape, disposed into the environment for groundwater recharge or supplement environmental flows, and to also meet municipal water demand (Adelodun et al. 2020a, b; Chang et al. 2020; Takeuchi and Tanaka 2020; Zhan et al. 2020). Furthermore, the generated sludge during the treatment of wastewaters could harbor some pollutants of PPCPs, which the analysis of such are not often carried out and could go undetected (Petrie et al. 2015), thereby leading to toxicity of the soil ecosystem and subsequently human health risk. For instance, some PPCP compounds like triclosan, ofloxacin, and ciprofloxacin have been found in the particulate phase of the final effluents in the range of 29–296 ng/l after secondary treatment processes of wastewater treatment plant (Petrie et al. 2014). Similarly, the comparative results of health risk and environmental impacts assessment of selected low mobile pharmaceuticals in biosolids amended to the soil in a regional European Union indicated that the mefenamic acid from the non-steroidal anti-inflammatory group had the highest ecological risk and environmental impact on aquatic biota, while sulfonamides and hydrochlorothiazide were moderately retained in the soil matrices (Morais et al. 2013). Thus, the reclaimed water needs to be sufficiently free from hazardous contaminants or below the permissible limits of various contaminants to avoid potential health risks to both humans and ecosystems (Adelodun et al. 2021a, b, c, d; Ajibade et al. 2021a, b).

The various treatment technology and removal methods targeted at the PPCPs require detailed comparison in terms of their level of treatment and removal of contaminants originating from the use of PPCPs. Moreover, the removal of the PPCPs from the wastewater treatment plants varied greatly from process to stage (Helbling et al. 2010), while some of the PPCPs persist for a longer period despite the long duration of digestion (Cortés et al. 2013). Thus, it is required to assess the health risk and ecotoxicity of using such treated wastewater for municipal, agriculture, or industrial processes, given the rising water scarcity challenges in many regions of the world due to climate change impact, and population growth, and urbanization. Kasprzyk-Hordern et al. investigated the treatment efficiency of two wastewater treatment plants with different treatment processes comprising activated sludge treatment and trickling filter beds (Kasprzyk-Hordern et al. 2009). The treatment plant which utilized the efficient activated sludge treatment had higher removal efficiency of over 85% for all the 55 PPCPs considered as compared to the treatment plant with trickling filter beds technology that resulted in less than 70% removal efficiency (Kasprzyk-Hordern et al. 2009). Nevertheless, the trickling filter beds technology was found to be highly effective with higher removal efficiency for p-benzylphenol, bisphenol A, and benzophenone-4. Nguyen et al. (2021) compared the effectiveness of different bioremediation techniques, including conventional activated sludge, membrane bioreactors, biofilm systems, and constructed wetlands under different operating conditions for selected PPCPs in wastewater treatment plants. The authors reported that biofilm systems of bioremediation, especially the hybrid process of moving bed biofilm reactor and integrated fixed-film activated sludge was highly efficient in the removal of a broad spectrum of PPCPs compounds in wastewater treatment plants as compared to other treatment techniques due to its acclimation of biomass, reduction in excess sludge production, and the metabolism of poorly degradable compounds (De La Torre et al. 2015; Nguyen et al. 2021).

Based on the previously reported studies on the limitation of photolysis, volatilization, and hydrolysis to significantly remove micropollutants in constructed wetlands, microbial degradation, substrate adsorption, and plant uptake were investigated as the primary pathways to remove the PPCPs in a constructed wetland. (Cheng et al. 2021) found that integrated microbial degradation, substrate adsorption, and plant uptake systems were highly effective in the removal of PPCPs with microbial degradation demonstrating the dominant pathway with a contribution of 86.69–99.95%. Fenton, Fenton-like (Liu et al. 2021), and ozonation (Wang and Chen 2020b) Paucar et al. (2019) processes have also been widely investigated. However, results or conclusions about the catalytic mechanisms are often inconsistent. The technologies involved in the removal of acetaminophen were also compared (Phong Vo et al. 2019), where ozonation indicated 100% removal efficiency of the acetaminophen as compared to chemical-based Fenton (87%), photo-based Fenton (84%), electro-based Fenton (96%), phytoremediation (64%), adsorption and filtration (98%), membrane (62%), and hybrid process (99%). Notwithstanding, the higher removal efficiency does not indicate total removal as there is a likelihood of transformation into less or more toxic metabolites, which could also be difficult to detect in environmental samples (Phong Vo et al. 2019). Ionizing radiation, also in combination with other methods, was also investigated (Wang and Chu 2016), showing that this could improve the degradation efficacy and reduce the treatment cost.

The environmental and operating conditions under which the treatment of various micropollutants occur also have a great influence on the degradation or treatment of PPCPs (Sui et al. 2015). For instance, chlortetracycline and tetracycline were degraded at a different rate under varying pH and temperature while degradation of sulfachlorpyridazine, sulfadimethoxine, sulfathiazole, and lincomycin were less influenced by changes in pH and temperature (Loftin et al. 2008). Furthermore, different operating conditions, including hydraulic and solid retention time, temperature, pH, and aerobic and anoxic processes were reported to enhance the biodegradation of compounds of PPCPs such as diclofenac, erythromycin, azithromycin, and clarithromycin, which are regarded as the priority PPCPs on the European Union watch list due to their recalcitrant nature and health risk on human and biota (Burke et al. 2014; Falås et al. 2013; Nguyen et al. 2021). The treatment of PPCPs in environmental samples using biological remediation has also been reported to be highly effective, especially when combined with the primary treatment process in the wastewater treatment plant (Rodriguez-Rodriguez et al. 2011).

The advanced wastewater treatment methods are of great significance to sustainably support the “3R” concept (reduce, reuse, and recycle) of wastewater management before they are discharged back into the environment. As a consequence of the population growth, waster stress is being experienced in and around urban areas because the sources of potable, clean freshwater are dwindling (Xiao et al. 2015). Therefore, reclaiming and reusing wastewater for other purposes is increasingly being practiced.

Table S7 (see supporting information) presents the selected treatment techniques of PPCPs along with the toxicity risk status of the effluent after treatment Ben et al. (2018) (Golet et al., 2002) Kosma et al. (2014).

**Fig. 2:**
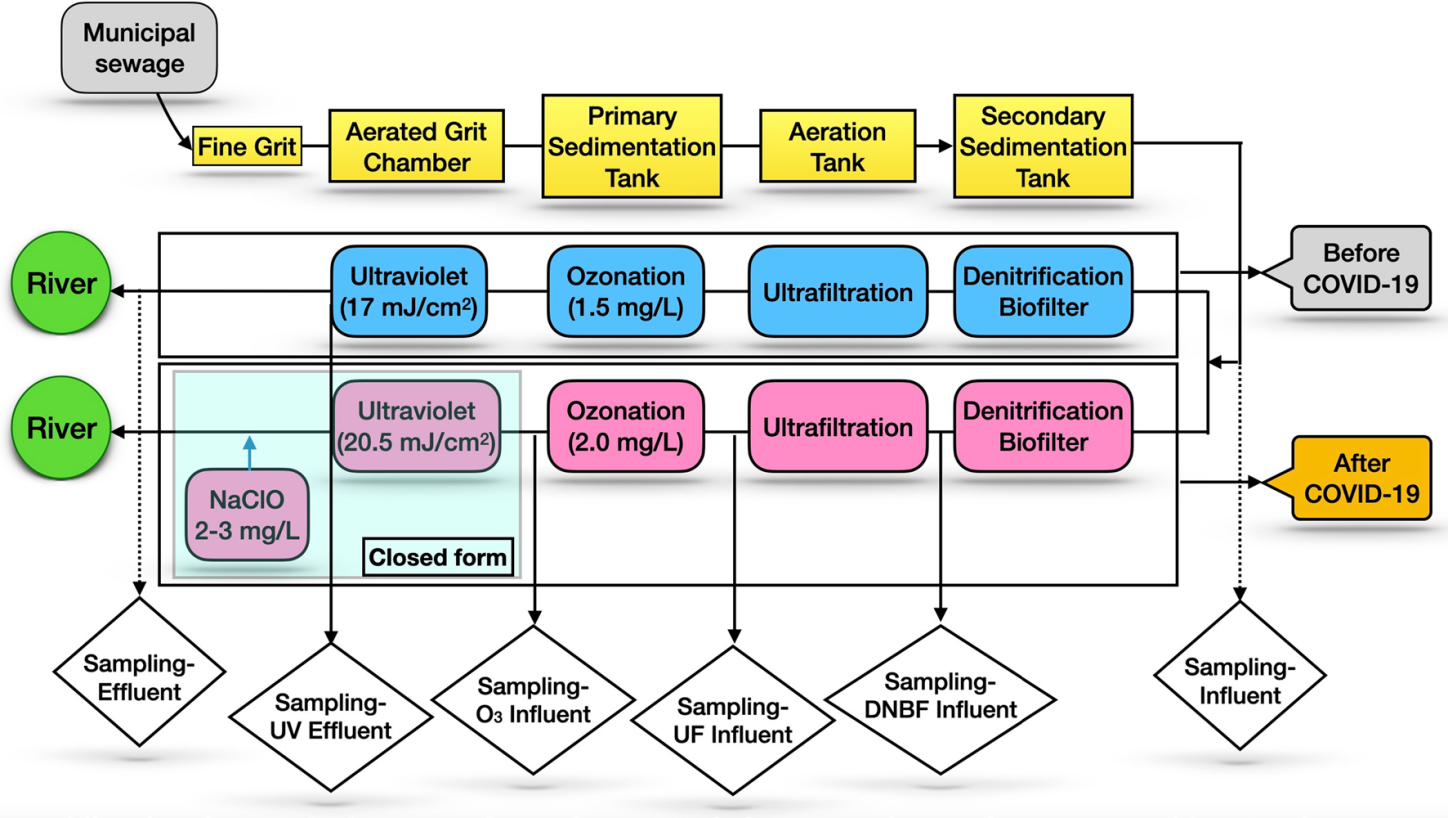
Reclaimed water treatment processes before and after the COVID-19 pandemic. Zhang et al. (2021) investigated a combined water treatment process, including both primary and secondary treatments. The additional treatment processes, which was incorporated due to the COVID-19, gave rise to a higher removal rate (> 80%) of the trace organic compounds from pharmaceutical and personal care products. Adapted from Zhang et al. (2021)

The previously developed treatment technologies and methods are likely to be inefficient for the PPCPs removal due to this latest development of the COVID-19 scenario. Zhang et al. investigated a combined water treatment process, including both primary and secondary treatments that considered the changes in operating conditions and different doses of disinfectants used before and after COVID-19 (Zhang et al. 2021) (Fig. 2). The authors found that the additional treatment processes incorporated due to the COVID-19 gave rise to a higher removal rate (> 80%) of the trace organic compounds from PPCPs, while chloroform (at < 15 μg/l) was the only resultant disinfection byproduct produced from the increased dose concentration of the chlorine (Zhang et al. 2021). The dosage of chlorine and the pH level has been reported to influence the removal of micropollutants during the chlorination process in the treatment plant (Nam et al. 2014).

## Perspective

The challenges and uncertainties associated with the presence of compounds of PPCPs and their potential risks in the ecosystem abound due to the arising need for the development of new PPCPs to combat the current COVID-19 pandemic and future ones. One of the major challenges with the management of the PPCPs in the wastewater treatment plant is the biotransformation of the parent compounds of some of the PPCPs rather than the intended removal or biodegradation. This process has been reported to increase the concentrations of some of the pollutants from the initial measured amount in the influents as compared to the effluents from the treatment plants (Nguyen et al. 2021; Zhang et al. 2017). Antibiotics (erythromycin, clarithromycin, azithromycin) and diclofenac are among the compounds that could deconjugate and then transform to the parent compounds via enzymatic activity or abiotic processes in the wastewater treatment plants (Nguyen et al. 2021; Vieno and Sillanpää 2014). Lee et al. found that equimolar diclofenac was formed within 7 days during the deconjugation of diclofenac ꞵ-O-acyl glucuronide (Lee et al. 2012). Moreover, the higher toxicity potential of the transformational products compared to the initial products like diclofenac and acetaminophen has been suggested by (Phong Vo et al. 2019; Schmitt-Jansen et al. 2007). There is a significant knowledge gap regarding the biotransformation of some of the PPCPs during the treatment process in wastewater treatment plants and the environment due to the lack of analytical methods and standard references (Basiuk et al. 2017; Nguyen et al. 2021; Petrie et al. 2015; Senta et al. 2019). Since there is a likelihood that the new conjugated compounds could possess some risks in the environment to which they are discharged (Nguyen et al. 2021), there is a need for more research on the transformation and potential risk assessment of various compounds of PPCPs in both the treatment plant and environment by expanding the monitoring capacities of more compounds in the environmental samples.

At present, the PPCPs have not been subjected to adequate monitoring in the environmental samples, with only a few countries, including the United States and the European Union currently having clear legislation and frameworks for the management of the micropollutants of PPCPs (Nguyen et al. 2021). Recently, the European Union listed 33 micropollutants that are most relevant to the wastewater treatment plants on the watch list (Decision 2015/495/EU) and required all the member states to monitor these substances at specific concentration benchmarks in the surface waters (Barbosa et al. 2016). One of the factors identified contributing to the monitoring problems of PPCPs in the environment is inadequate sampling strategies, especially for the unregulated PPCPs (Petrie et al. 2015). The popular discrete grab method is limited in identifying the concentration of pollutants at a specific point in time. However, the time or flow proportional composite sampling that could address the fluctuations inflow is relatively less adopted due to the associated high cost and logistic constraints (Coutu et al. 2013; Petrie et al. 2015; Plósz et al. 2010). An integrated analytical approach that could be deployed to assess the toxicity of targeted and non-targeted micropollutants distribution, both spatially and temporally in the environmental media is suggested to ensure accurate risk assessment (Petrie et al. 2015).

The particulate phase analysis of PPCPs in wastewater is another identified area that has not been thoroughly implemented in the literature to monitor the performance of the various technologies involved in the treatment plants which could, however, assist to understand the pathways of the micropollutants removals during the wastewater treatment process (Petrie et al. 2014). The integration of both the commonly used aqueous phase and the particulate phase analyses for each sampling point would provide a complete mass balance process and an understanding of the dominant mechanisms involved in the treatment process (Petrie et al. 2015). The knowledge of the micropollutants removal process and their fate in the wastewater treatment plants could provide the needed information for further research on the optimization of the treatment process for optimum removal efficiency.

**Fig. 3:**
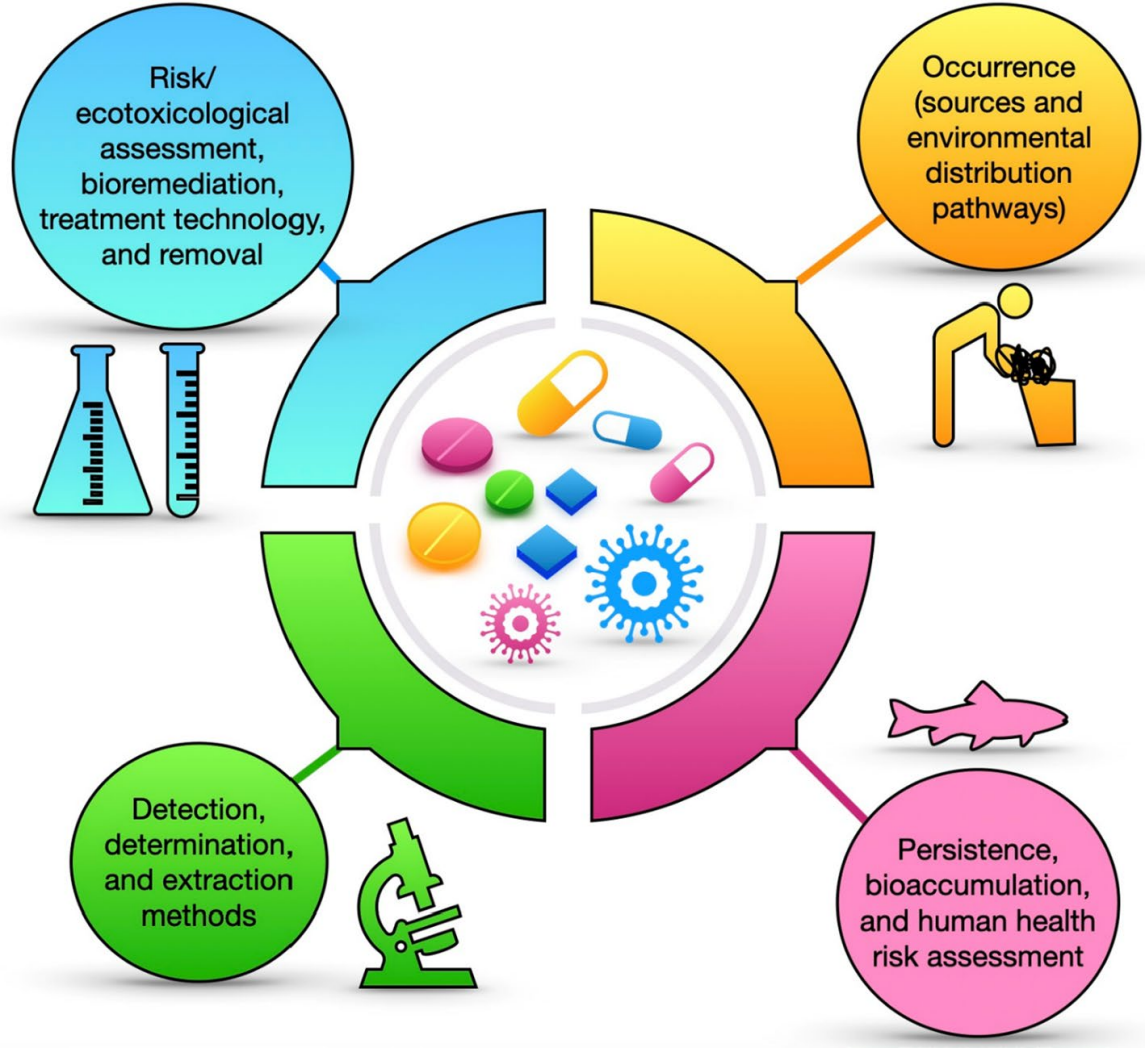
Pharmaceuticals and personal care products physicochemical characteristics make them potentially dangerous for aquatic organisms and human health. Despite the recent advances in analytical techniques that allow to highlight the environmental presence of such chemicals, there are some gaps in the state of knowledge of pharmaceuticals and personal care products presence. Then, legislation on minimum levels of pharmaceuticals and personal care products allowed to be released into the environment should be implemented also for treated effluents and sludge used as a fertilizer

Furthermore, there should be institutional legislation and guidelines on the use of treated effluents and sludge containing the micropollutants of PPCPs as fertilizers for soil amendments and likewise the safe concentration discharge into the environment (Goala et al. 2021). Many countries, especially from the developing and less developed regions do not have specific laws guiding the use of effluents and sludge as regards the PPCPs concentrations for soil amendment, thereby making the population in those regions vulnerable to the health risk of the PPCPs contaminants. Moreover, there is a need for more studies on the environmental risk assessment of transformed and conjugated products of PPCPs from the effluents of the treatment plants to forestall any potential health risk to humans and other biota in the environment (Fig. 3).

## Supplementary Information

Below is the link to the electronic supplementary material.

Supplementary file 1 (DOCX 2062 kb)

## Data Availability

Not applicable.

## References

[CR1] Adams C, Wang Y, Loftin K, Meyer M (2002). Removal of antibiotics from surface and distilled water in conventional water treatment processes. J Environ Eng.

[CR2] Adelodun B, Ajibade FO, Ogunshina MS, Choi K-S (2019). Dosage and settling time course optimization of Moringa oleifera in municipal wastewater treatment using response surface methodology. Desalin Water Treat.

[CR3] Adelodun B, Ajibade FO, Ibrahim RG, Bakare HO, Choi K-S (2020). Snowballing transmission of COVID-19 (SARS-CoV-2) through wastewater: any sustainable preventive measures to curtail the scourge in low-income countries?. Sci Total Environ.

[CR4] Adelodun B, Ogunshina MS, Ajibade FO, Abdulkadir TS, Bakare HO, Choi KS (2020). Kinetic and prediction modeling studies of organic pollutants removal from municipal wastewater using moringa oleifera biomass as a coagulant. Water.

[CR5] Adelodun B, Ajibade FO, Ibrahim RG, Ighalo JO, Bakare HO, Kumar P, Eid EM, Kumar V, Odey G, Choi KS (2021). Insights into hazardous solid waste generation during COVID-19 pandemic and sustainable management approaches for developing countries. J Mater Cycles Waste Manag.

[CR6] Adelodun B, Ajibade FO, Ighalo JO, Odey G, Ibrahim RG, Kareem KY, Bakare HO, Tiamiyu AO, Ajibade TF, Abdulkadir TS, Adeniran KA, Choi KS (2021). Assessment of socioeconomic inequality based on virus-contaminated water usage in developing countries: a review. Environ Res.

[CR7] Adelodun B, Ajibade FO, Tiamiyu AO, Nwogwu NA, Ibrahim RG, Kumar P, Kumar V, Odey G, Yadav KK, Khan AH, Cabral-Pinto MMS, Kareem KY, Bakare HO, Ajibade TF, Naveed QN, Islam S, Fadare OO, Choi KS (2021). Monitoring the presence and persistence of SARS-CoV-2 in water-food-environmental compartments: state of the knowledge and research needs. Environ Res.

[CR8] Adelodun B, Tiamiyu AO, Ajibade FO, Odey G, Ibrahim RG, Goala M, Bakare HO, Ajibade TF, Adeniran JA, Adeniran KA, Choi KS, Dehghani MH, Karri RR, Roy S (2021). Presence, detection, and persistence of SARS-CoV-2 in wastewater and the sustainable remedial measures. Environmental and health management of novel coronavirus disease (COVID-19).

[CR9] Agüera A, Pérez Estrada LA, Ferrer I, Thurman EM, Malato S, Fernández-Alba AR (2005). Application of time-of-flight mass spectrometry to the analysis of phototransformation products of diclofenac in water under natural sunlight. J Mass Spectrom.

[CR10] Aitken M (2020). Shifts in healthcare demand, delivery and care during the COVID-19 era.

[CR11] Ajibade FO, Adelodun B, Lasisi KH, Fadare OO, Ajibade TF, Nwogwu NA, Sulaymon ID, Ugya AY, Wang HC, Wang A, Oladoja NA, Unuabonah EI (2021). Environmental pollution and their socioeconomic impacts. Microbe mediated remediation of environmental contaminants.

[CR12] Ajibade FO, Nwogwu NA, Lasisi KH, Ajibade TF, Adelodun B, Guadie A, Ugya AY, Adewumi JR, Wang HC, Wang A, Oladoja NA, Unuabonah EI (2021). Progress and prospects in the management of oxyanion polluted aqua systems. Removal of nitrogen oxyanion (nitrate) in constructed wetlands.

[CR13] Anand U, Cabreros C, Mal J, Ballesteros F, Sillanpää M, Tripathi V, Bontempi E (2021). Novel coronavirus disease 2019 (COVID-19) pandemic: from transmission to control with an interdisciplinary vision. Environ Res.

[CR14] Anand U, Adelodun B, Pivato A, Suresh S, Indari O, Jakhmola S, Jha HC, Jha PK, Tripathi V, Di Maria F (2021). A review of the presence of SARS-CoV-2 RNA in wastewater and airborne particulates and its use for virus spreading surveillance. Environ Res.

[CR15] Anand U, Jakhmola S, Indari O, Chandra Jha H, Chen ZS, Tripathi V, Pérez de la Lastra JM (2021). Potential therapeutic targets and vaccine development for COVID-19 management: a review on the recent update. Front Immunol.

[CR16] Anand U, Reddy B, Singh VK, Singh AK, Kesari KK, Tripathi P, Kumar P, Tripathi V, Simal-Gandara J (2021). Potential environmental and human health risks caused by antibiotic-resistant bacteria (ARB), antibiotic resistance genes (ARGs) and emerging contaminants (ECs) from municipal solid waste (MSW) landfill. Antibiotics.

[CR17] Anand U, Li X, Sunita K, Lokhandwala S, Gautam P, Suresh S, Sarma H, Vellingiri B, Dey A, Bontempi E, Jiang G (2022). SARS-CoV-2 and other pathogens in municipal wastewater, landfill leachate, and solid waste: a review about virus surveillance, infectivity, and inactivation. Environ Res.

[CR18] Andersen KG, Rambaut A, Lipkin WI, Holmes EC, Garry RF (2020). The proximal origin of SARS-CoV-2. Nat Med.

[CR19] Arismendi D, Becerra-Herrerab M, Cerratoa I, Richtera P (2019). Simultaneous determination of multiresidue and multiclass emerging contaminants in waters by rotating-disk sorptive extraction-derivatization gas chromatography/mass spectrometry. Talanta.

[CR20] Arnnok P, Singh RR, Burakham R, Pérez-Fuentetaja A, Aga DS (2017). Selective uptake and bioaccumulation of antidepressants in fish from effluent-impacted Niagara river. Environ Sci Technol.

[CR21] Ashfaq M, Li Y, Wang Y, Chen W, Wang H, Chen X, Wu W, Huang Z, Yu CP, Sun Q (2017). Occurrence, fate, and mass balance of different classes of pharmaceuticals and personal care products in an anaerobic–anoxic–oxic wastewater treatment plant in Xiamen, China. Water Res.

[CR22] Awfa D, Ateia M, Fujii M, Yoshimura C (2019). Novel magnetic carbon nanotube-TiO_2_ composites for solar light photocatalytic degradation of pharmaceuticals in the presence of natural organic matter. J Water Process Eng.

[CR23] Ayati N, Saiyarsarai P, Nikfar S (2020). Short and long term impacts of COVID-19 on the pharmaceutical sector. DARU J Pharm Sci.

[CR24] Bachmann K, Hacker M, Messer W, Bachmann K (2009). Drug metabolism. Pharmacology.

[CR25] Balakrishna K, Rath A, Praveenkumarreddy Y, Guruge KS, Subedi B (2017). A review of the occurrence of pharmaceuticals and personal care products in Indian water bodies. Ecotoxicol Environ Saf.

[CR26] Bandala ER, Rodriguez-Narvaez OM (2019). On the nature of hydrodynamic cavitation process and its application for the removal of water pollutants. Air Soil Water Res.

[CR27] Bandala ER, Kruger BR, Cesarino I, Leao AL, Wijesiri B, Goonetilleke A (2021). Impacts of COVID-19 pandemic on the wastewater pathway into surface water: a review. Sci Total Environ.

[CR28] Barbosa MO, Moreira NFF, Ribeiro AR, Pereira MFR, Silva AMT (2016). Occurrence and removal of organic micropollutants: an overview of the watch list of EU Decision 2015/495. Water Res.

[CR29] Basaglia G, Pietrogrande MC (2012). Optimization of a SPME/GC/MS method for the simultaneous determination of pharmaceuticals and personal care products in waters. Chromatographia.

[CR30] Basiuk M, Brown RA, Cartwright D, Davison R, Wallis PM (2017). Trace organic compounds in rivers, streams, and wastewater in southeastern Alberta, Canada. Inland Waters.

[CR31] Batt AL, Kim S, Aga DS (2007). Comparison of the occurrence of antibiotics in four full-scale wastewater treatment plants with varying designs and operations. Chemosphere.

[CR32] Bayati M, Ho TL, Vu DC, Wang F, Rogers E, Cuvellier C, Huebotter S, Inniss EC, Udawatta R, Jose S, Lin CH (2021). Assessing the efficiency of constructed wetlands in removing PPCPs from treated wastewater and mitigating the ecotoxicological impacts. Int J Hygiene Environ Health.

[CR33] Ben W, Zhu B, Yuan X, Zhang Y, Yang M, Qiang Z (2018). Occurrence, removal and risk of organic micropollutants in wastewater treatment plants across China: comparison of wastewater treatment processes. Water Res.

[CR34] Benotti MJ, Stanford BD, Wert EC, Snyder SA (2009). Evaluation of a photocatalytic reactor membrane pilot system for the removal of pharmaceuticals and endocrine disrupting compounds from water. Water Res.

[CR35] Binti-Muhamad M, Binti-Mohamed-Zuki F (2020). Attitude and perception on the disposal of pharmaceuticals and personal care products in malaysia: a pilot study. IOP Conf Ser Mater Sci Eng.

[CR36] BIO Intelligence Service (2013) Study on the environmental risks of medicinal products. Final report prepared for Executive Agency for Health and Consumers (Issue December)

[CR37] Bong CPC, Goh RKY, Lim JS, Ho WS, Lee CT, Hashim H, Abu Mansor NN, Ho CS, Ramli AR, Takeshi F (2017). Towards low carbon society in Iskandar Malaysia: implementation and feasibility of community organic waste composting. J Environ Manag.

[CR38] Bontempi E, Sorrentino GP, Zanoletti A, Alessandri I, Depero LE, Caneschi A (2021). Sustainable materials and their contribution to the sustainable development goals (SDGs): a critical review based on an Italian example. Molecules.

[CR39] Bookwalter CM (2021). Drug shortages amid the COVID-19 pandemic. US Pharm.

[CR40] Bratkowska D, Marcé RM, Cormack PAG, Borrull F, Fontanals N (2011). Development and application of a polar coating for stir bar sorptive extraction of emerging pollutants from environmental water samples. Anal Chim Acta.

[CR41] Burke V, Richter D, Hass U, Duennbier U, Greskowiak J, Massmann G (2014). Redox-dependent removal of 27 organic trace pollutants: compilation of results from tank aeration experiments. Environ Earth Sci.

[CR42] Burkina V, Zlabek V, Zamaratskaia G (2015). Effects of pharmaceuticals present in aquatic environment on Phase I metabolism in fish. Environ Toxicol Pharmacol.

[CR43] Burns EE, Carter LJ, Kolpin DW, Thomas-Oates J, Boxall ABA (2018). Temporal and spatial variation in pharmaceutical concentrations in an urban river system. Water Res.

[CR44] Caldas SS, Rombaldi C, de Oliveira Arias JL, Marube LC, Primel EG (2016). Multi-residue method for determination of 58 pesticides, pharmaceuticals and personal care products in water using solvent demulsification dispersive liquid–liquid microextraction combined with liquid chromatography-tandem mass spectrometry. Talanta.

[CR45] Carvalho PN, Araújo JL, Mucha AP, Basto MCP, Almeida CMR (2013). Potential of constructed wetlands microcosms for the removal of veterinary pharmaceuticals from livestock wastewater. Biores Technol.

[CR46] Chang N, Zhang Q, Wang Q, Luo L, Wang XC, Xiong J, Han J (2020). Current status and characteristics of urban landscape lakes in China. Sci Total Environ.

[CR47] Chen F, Gong Z, Kelly BC (2017). Bioaccumulation behavior of pharmaceuticals and personal care products in adult Zebrafish (*Danio rerio*): influence of physical–chemical properties and biotransformation. Environ Sci Technol.

[CR48] Chen Z, Guo J, Jiang Y, Shao Y (2021). High concentration and high dose of disinfectants and antibiotics used during the COVID-19 pandemic threaten human health. Environ Sci Eur.

[CR49] Cheng YX, Chen J, Wu D, Liu YS, Yang YQ, He LX, Ye P, Zhao JL, Liu SS, Yang B, Ying GG (2021). Highly enhanced biodegradation of pharmaceutical and personal care products in a novel tidal flow constructed wetland with baffle and plants. Water Res.

[CR50] Cheraghali AM (2017). Trends in Iran pharmaceutical market. Iran J Pharm Res IJPR.

[CR51] Clarke BO, Anumol T, Barlaz M, Snyder SA (2015). Investigating landfill leachate as a source of trace organic pollutants. Chemosphere.

[CR52] Collado N, Rodriguez-Mozaz S, Gros M, Rubirola A, Barceló D, Comas J, Rodriguez-Roda I, Buttiglieri G (2014). Pharmaceuticals occurrence in a WWTP with significant industrial contribution and its input into the river system. Environ Pollut.

[CR53] Commission E (2019). Communication from the Commission to the European Parliament, the Council, and the European Economic and Social Committee: European union strategic approach to pharmaceuticals in the environment. EU Commision.

[CR54] Cortés JM, Larsson E, Jönsson JÅ (2013). Study of the uptake of non-steroid anti-inflammatory drugs in wheat and soybean after application of sewage sludge as a fertilizer. Sci Total Environ.

[CR55] Cory WC, Welch AM, Ramirez JN, Rein LC (2019). Naproxen and Its phototransformation products: persistence and ecotoxicity to toad tadpoles (*Anaxyrus terrestris*), individually and in mixtures. Environ Toxicol Chem.

[CR56] Costa-Rama E, Nouws HPA, Delerue-Matos C, Blanco-López MC, Fernández-Abedul MT (2020) Electrochemical sensors for emerging contaminants: diclofenac preconcentration and detection on paper-based electrodes. In: Advances in science, technology and innovation (Issue November), pp 227–229. 10.1007/978-3-030-13068-8_56

[CR57] Coutu S, Wyrsch V, Wynn HK, Rossi L, Barry DA (2013). Temporal dynamics of antibiotics in wastewater treatment plant influent. Sci Total Environ.

[CR58] Dai G, Huang J, Chen W, Wang B, Yu G, Deng S (2014). Major pharmaceuticals and personal care products (PPCPs) in wastewater treatment plant and receiving water in Beijing, China, and associated ecological risks. Bull Environ Contam Toxicol.

[CR59] De La Torre T, Alonso E, Santos JL, Rodríguez C, Gómez MA, Malfeito JJ (2015). Trace organics removal using three membrane bioreactor configurations: MBR, IFAS-MBR and MBMBR. Water Sci Technol.

[CR60] de Solla SR, Gilroy ÈAM, Klinck JS, King LE, McInnis R, Struger J, Backus SM, Gillis PL (2016). Bioaccumulation of pharmaceuticals and personal care products in the unionid mussel *Lasmigona costata* in a river receiving wastewater effluent. Chemosphere.

[CR61] Desgens-Martin V, Keller AA (2021). COVID-19 treatment agents: do they pose an environmental risk?. ACS ES&T Water.

[CR62] Dewey HM, Jones JM, Keating MR, Budhathoki-Uprety J (2021). Increased use of disinfectants during the COVID-19 pandemic and its potential impacts on health and safety. ACS Chem Health Saf.

[CR220] Daughton C (2004) Non-regulated water contaminants: emerging research - Environ. Impact Assessment Rev., 24: pp. 711-732. 10.1016/j.eiar.2004.06.003

[CR63] Ebele AJ, Abou-Elwafa Abdallah M, Harrad S (2017). Pharmaceuticals and personal care products (PPCPs) in the freshwater aquatic environment. Emerg Contam.

[CR64] Esseku YY (2016) Drug disposal flow diagrams and sustainable water quality. In: 39th WEDC international conference, Kumasi, Ghana, 2016 ensuring. Ensuring availability and sustainable management of water and sanitation for all

[CR65] Fahimi A, Zanoletti A, Federici S, Assi A, Bilo F, Depero LE, Bontempi E (2020). New eco-materials derived from waste for emerging pollutants adsorption: the case of diclofenac. Materials.

[CR66] Falås P, Longrée P, La Cour Jansen J, Siegrist H, Hollender J, Joss A (2013). Micropollutant removal by attached and suspended growth in a hybrid biofilm-activated sludge process. Water Res.

[CR67] Fang W, Peng Y, Muir D, Lin J, Zhang X (2019). A critical review of synthetic chemicals in surface waters of the US, the EU and China suppl. Environ Int.

[CR68] FAO/WHO (2009) FAO Fisheries and Aquaculture Report No. 978 report of the joint FAO/WHO expert consultation on the risks and benefits of fish consumption

[CR69] Fenech C, Nolan K, Rock L, Morrissey A (2013). An SPE LC-MS/MS method for the analysis of human and veterinary chemical markers within surface waters: an environmental forensics application. Environ Pollut.

[CR70] Franklin AM, Williams CF, Andrews DM, Woodward EE, Watson JE (2016). Uptake of three antibiotics and an antiepileptic drug by wheat crops spray irrigated with wastewater treatment plant effluent. J Environ Qual.

[CR71] Frédéric O, Yves P (2014). Pharmaceuticals in hospital wastewater: their ecotoxicity and contribution to the environmental hazard of the effluent. Chemosphere.

[CR72] Garrison AW, Pope JD, Allen FR, Keith LH (1976). GC/MS analysis of organic compounds in domestic wastewaters. Identification and analysis of organic pollutants in water.

[CR73] Ghafoor D, Khan Z, Khan A, Ualiyeva D, Zaman N (2021). Excessive use of disinfectants against COVID-19 posing a potential threat to living beings. Curr Res Toxicol.

[CR74] Gilart N, Núria G, Marcé RM, Borrull F, Fontanals N (2013). Novel coatings for stir bar sorptive extraction to determine pharmaceuticals and personal care products in environmental waters by liquid chromatography and tandem mass spectrometry. Anal Chim Acta.

[CR75] Goala M, Yadav KK, Alam J, Adelodun B, Choi KS, Cabral-Pinto MMS, Hamid AA, Alhoshan M, Ali FAA, Shukla AK (2021). Phytoremediation of dairy wastewater using *Azolla pinnata*: application of image processing technique for leaflet growth simulation. J Water Process Eng.

[CR76] Golet EM, Alder AC, Giger W (2002). Environmental exposure and risk assessment of fluoroquinolone antibacterial agents in wastewater and river water of the Glatt Valley watershed, Switzerland. Environ Sci Technol.

[CR77] Gottschall N, Topp E, Metcalfe C, Edwards M, Payne M, Kleywegt S, Russell P, Lapen DR (2012). Pharmaceutical and personal care products in groundwater, subsurface drainage, soil, and wheat grain, following a high single application of municipal biosolids to a field. Chemosphere.

[CR78] Guerra P, Kim M, Shah A, Alaee M, Smyth SA (2014). Occurrence and fate of antibiotic, analgesic/anti-inflammatory, and antifungal compounds in five wastewater treatment processes. Sci Total Environ.

[CR79] Hamscher G, Sczesny S, Höper H, Nau H (2002). Determination of persistent tetracycline residues in soil fertilized with liquid manure by high-performance liquid chromatography with electrospray ionization tandem mass spectrometry. Anal Chem.

[CR80] Harrison EZ, Oakes SR, Hysell M, Hay A (2006). Organic chemicals in sewage sludges. Sci Total Environ.

[CR81] He Y, Sutton NB, Rijnaarts HHH, Langenhoff AAM (2016). Degradation of pharmaceuticals in wastewater using immobilized TiO2 photocatalysis under simulated solar irradiation. Appl Catal B.

[CR82] Heidler J, Sapkota A, Halden RU (2006). Partitioning, persistence, and accumulation in digested sludge of the topical antiseptic triclocarban during wastewater treatment. Environ Sci Technol.

[CR83] Helbling DE, Hollender J, Kohler HPE, Singer H, Fenner K (2010). High-throughput identification of microbial transformation products of organic micropollutants. Environ Sci Technol.

[CR84] Hignite C, Azarnoff DL (1977). Drugs and drug metabolites as environmental contaminants: chlorophenoxyisobutyrate and salicylic acid in sewage water effluent. Life Sci.

[CR221] Hoenicke R, Oros DR, Oram JJ, Taberski KM (2007) Adapting an ambient monitoring program to the challenge of managing emerging pollutants in the San Francisco Estuary Environmental Research. 105(1): 132–144. 10.1016/j.envres.2007.01.005 10.1016/j.envres.2007.01.005 17336284

[CR85] Huerta B, Jakimska A, Gros M, Rodriguez-Mozaz S, Barcelo D (2013). Analysis of multi-class pharmaceuticals in fish tissues by ultra-high-performance liquid chromatography tandem mass spectrometry. J Chromatogr A.

[CR86] Ibrahimagić O, Ercegović Z, Vujadinović A, Kunić S (2020). Comment on an article: “Medications in COVID-19 patients: summarizing the current literature from an orthopaedic perspective”. Int Orthop.

[CR87] Iglesias A, Nebot C, Vázquez BI, Miranda JM, Abuín CMF, Cepeda A (2014). Detection of veterinary drug residues in surface waters collected nearby farming areas in Galicia, North of Spain. Environ Sci Pollut Res.

[CR88] Iyer M, Tiwari S, Renu K, Pasha MY, Pandit S, Singh B, Raj N, Krothapalli S, Kwak HJ, Balasubramanian V, Jang SB (2021). Environmental survival of SARS-CoV-2—a solid waste perspective. Environ Res.

[CR89] Kanama KM, Daso AP, Mpenyana-Monyatsi L, Coetzee MAA (2018). Assessment of pharmaceuticals, personal care products, and hormones in wastewater treatment plants receiving inflows from health facilities in North West Province, South Africa. J Toxicol.

[CR90] Kareem KY, Adelodun B, Tiamiyu AO, Ajibade FO, Ibrahim RG, Odey G, Goala M, Bakare HO, Adeniran JA, Hussain CM, Shukla SK (2021). Effects of COVID-19: an environmental point of view. Detection and analysis of SARS coronavirus.

[CR91] Kasprzyk-Hordern B, Dinsdale RM, Guwy AJ (2009). The removal of pharmaceuticals, personal care products, endocrine disruptors and illicit drugs during wastewater treatment and its impact on the quality of receiving waters. Water Res.

[CR92] Kassie AD, Bifftu BB, Mekonnen HS (2018). Self-medication practice and associated factors among adult household members in Meket district, Northeast Ethiopia, 2017. BMC Pharmacol Toxicol.

[CR93] Keerthanan S, Jayasinghe C, Biswas JK, Vithanage M (2021). Pharmaceutical and Personal Care Products (PPCPs) in the environment: plant uptake, translocation, bioaccumulation, and human health risks. Crit Rev Environ Sci Technol.

[CR94] Kim I, Tanaka H (2009). Photodegradation characteristics of PPCPs in water with UV treatment. Environ Int.

[CR95] Kim HJ, Lee DS, Kwon JH (2010). Sorption of benzimidazole anthelmintics to dissolved organic matter surrogates and sewage sludge. Chemosphere.

[CR96] Kolpin DW, Furlong ET, Meyer MT, Thurman EM, Zaugg SD, Barber LB, Buxton HT (2002). Pharmaceuticals, hormones, and other organic wastewater contaminants in U.S. streams, 1999–2000: a national reconnaissance. Environ Sci Technol.

[CR97] Kosjek T, Heath E (2008). Applications of mass spectrometry to identifying pharmaceutical transformation products in water treatment. TrAC Trends Anal Chem.

[CR98] Kosma CI, Lambropoulou DA, Albanis TA (2014). Investigation of PPCPs in wastewater treatment plants in Greece: occurrence, removal and environmental risk assessment. Sci Total Environ.

[CR223] Kotnik K, Kosjek T, Krajnc U, Heath E (2014) Trace analysis of benzophenone derived compounds in surface waters and sediments using solid-phase extraction and microwave-assisted extraction followed by gas chromatography-mass spectrometry. Anal. Bioanal. Chem. 406 (13): 3179–3190. 10.1007/s00216-014-7749-0 24682231

[CR99] Krzeminski P, Tomei MC, Karaolia P, Langenhoff A, Almeida CMR, Felis E, Gritten F, Andersen HR, Fernandes T, Manaia CM, Rizzo L, Fatta-Kassinos D (2019). Performance of secondary wastewater treatment methods for the removal of contaminants of emerging concern implicated in crop uptake and antibiotic resistance spread: a review. Sci Total Environ.

[CR100] Kumar R, Sarmah AK, Padhye LP (2019). Fate of pharmaceuticals and personal care products in a wastewater treatment plant with parallel secondary wastewater treatment train. J Environ Manag.

[CR101] Kumar M, Kuroda K, Dhangar K, Mazumder P, Sonne C, Rinklebe J, Kitajima M (2020). Potential emergence of antiviral-resistant pandemic viruses via environmental drug exposure of animal reservoirs. Environ Sci Technol.

[CR102] Kuzmanović M, López-Doval JC, De Castro-Català N, Guasch H, Petrović M, Muñoz I, Ginebreda A, Barceló D (2016). Ecotoxicological risk assessment of chemical pollution in four Iberian river basins and its relationship with the aquatic macroinvertebrate community status. Sci Total Environ.

[CR103] Lee HJ, Lee E, Yoon SH, Chang HR, Kim K, Kwon JH (2012). Enzymatic and microbial transformation assays for the evaluation of the environmental fate of diclofenac and its metabolites. Chemosphere.

[CR104] Lei K, Zhou Y, Chen W, Pan HY, Guo BB, Zhang X, Cao YX, Sweetman AJ, Lin CY (2018). The occurrence of home and personal care products in the Haihe River catchment and estimation of human exposure. Sci Total Environ.

[CR105] Li S, Shi WZ, Li HM, Xu N, Zhang RJ, Chen XJ (2018). Antibiotics in water and sediments of rivers and coastal area of Zhuhai City, Pearl River estuary, south China. Sci Total Environ.

[CR106] Li Y, Niu X, Yao C, Yang W, Lu G (2019). Distribution, removal, and risk assessment of pharmaceuticals and their metabolites in five sewage plants. Int J Environ Res Public Health.

[CR107] Lin H, Li H, Chen L, Li L, Yin L, Lee H, Yang Z (2018). Mass loading and emission of thirty-seven pharmaceuticals in a typical municipal wastewater treatment plant in Hunan Province, Southern China. Ecotoxicol Environ Saf.

[CR108] Lin X, Xu J, Keller AA, He L, Gu Y, Zheng W, Sun D, Lu Z, Huang J, Huang X, Li G (2020). Occurrence and risk assessment of emerging contaminants in a water reclamation and ecological reuse project. Sci Total Environ.

[CR109] Lindberg RH, Wennberg P, Johansson MI, Tysklind M, Andersson BAV (2005). Screening of human antibiotic substances and determination of weekly mass flows in five sewage treatment plants in Sweden. Environ Sci Technol.

[CR110] Liu Y, Zhao Y, Wang J (2021). Fenton/Fenton-like processes with in-situ production of hydrogen peroxide/hydroxyl radical for degradation of emerging contaminants: advances and prospects. J Hazard Mater.

[CR111] Loftin KA, Adams CD, Meyer MT, Surampalli R (2008). Effects of ionic strength, temperature, and pH on degradation of selected antibiotics. J Environ Qual.

[CR112] Lorenzetti AS, Sierra T, Domini CE, Lista AG, Crevillen AG, Escarpa A (2020). Electrochemically reduced graphene oxide-based screen-printed electrodes for total tetracycline determination by adsorptive transfer stripping differential pulse voltammetry. Sensors (switzerland).

[CR113] Lozano N, Rice CP, Ramirez M, Torrents A (2012). Fate of triclosan and methyltriclosan in soil from biosolids application. Environ Pollut.

[CR114] Luo S, Wei Z, Spinney R, Zhang Z, Dionysiou DD, Gao L, Chai L, Wang D, Xiao R (2018). UV direct photolysis of sulfamethoxazole and ibuprofen: an experimental and modelling study. J Hazard Mater.

[CR115] Malik M, Tahir MJ, Jabbar R, Ahmed A, Hussain R (2020). Self-medication during Covid-19 pandemic: challenges and opportunities. Drugs Ther Perspect.

[CR116] Mani A, Thawani V (2019). The persisting environmental problem of disposal of expired and unused medicines. J Mahatma Gandhi Inst Med Sci.

[CR117] Marasco Júnior CA, Luchiari NDC, Lima Gomes PCF (2019). Occurrence of caffeine in wastewater and sewage and applied techniques for analysis: a review. Eclét Quím J.

[CR118] Matongo S, Birungi G, Moodley B, Ndungu P (2015). Pharmaceutical residues in water and sediment of Msunduzi River, KwaZulu-Natal, South Africa. Chemosphere.

[CR119] Medrano-Rodríguez F, Picos-Benítez A, Brillas E, Bandala ER, Pérez T, Peralta-Hernández JM (2020). Electrochemical advanced oxidation discoloration and removal of three brown diazo dyes used in the tannery industry. J Electroanal Chem.

[CR120] Meng Y, Liu W, Liu X, Zhang J, Peng M, Zhang T (2021). A review on analytical methods for pharmaceutical and personal care products and their transformation products. J Environ Sci (china).

[CR121] Meyer MF, Powers SM, Hampton SE (2019). An evidence synthesis of pharmaceuticals and personal care products (PPCPs) in the environment: imbalances among compounds, sewage treatment techniques, and ecosystem types. Environ Sci Technol.

[CR122] Miyazaki A, Tung R, Taing B, Matsui M, Iwamoto A, Cox SE (2020). Frequent unregulated use of antibiotics in rural Cambodian infants. Trans R Soc Trop Med Hyg.

[CR123] Mohapatra DP, Kirpalani DM (2019). Advancement in treatment of wastewater: fate of emerging contaminants. Can J Chem Eng.

[CR124] Mohapatra S, Huang CH, Mukherji S, Padhye LP (2016). Occurrence and fate of pharmaceuticals in WWTPs in India and comparison with a similar study in the United States. Chemosphere.

[CR125] Mompelat S, Le Bot B, Thomas O (2009). Occurrence and fate of pharmaceutical products and by-products, from resource to drinking water. Environ Int.

[CR222] Mons MN, Hoogenboom AC, Noij THM (2003) Pharmaceuticals and drinking water supply in the Netherlands. Nieuwegein, Kiwa Water Research, Nieuwegein, The Netherlands.

[CR126] Morais SA, Delerue-Matos C, Gabarrell X, Blánquez P (2013). Multimedia fate modeling and comparative impact on freshwater ecosystems of pharmaceuticals from biosolids-amended soils. Chemosphere.

[CR127] Muir D, Simmons D, Wang X, Peart T, Villella M, Miller J, Sherry J (2017). Bioaccumulation of pharmaceuticals and personal care product chemicals in fish exposed to wastewater effluent in an urban wetland. Sci Rep.

[CR128] Mutiyar PK, Mittal AK (2013). Occurrences and fate of an antibiotic amoxicillin in extended aeration-based sewage treatment plant in Delhi, India: a case study of emerging pollutant. Desalin Water Treat.

[CR129] Mutiyar PK, Mittal AK (2014). Occurrences and fate of selected human antibiotics in influents and effluents of sewage treatment plant and effluent-receiving river Yamuna in Delhi (India). Environ Monit Assess.

[CR130] Mutiyar PK, Gupta SK, Mittal AK (2018). Fate of pharmaceutical active compounds (PhACs) from River Yamuna, India: an ecotoxicological risk assessment approach. Ecotoxicol Environ Saf.

[CR131] Nam SW, Jo BI, Yoon Y, Zoh KD (2014). Occurrence and removal of selected micropollutants in a water treatment plant. Chemosphere.

[CR132] Nazari E, Suja F (2016). Effects of 17β-estradiol (E2) on aqueous organisms and its treatment problem: a review. Rev Environ Health.

[CR133] Nguyen PY, Carvalho G, Reis MAM, Oehmen A (2021). A review of the biotransformations of priority pharmaceuticals in biological wastewater treatment processes. Water Res.

[CR134] OECD (2019) Pharmaceutical residues in freshwater. OECD studies on water, 136

[CR135] Ort C, Lawrence MG, Reungoat J, Mueller JF (2010). Sampling for PPCPs in wastewater systems: comparison of different sampling modes and optimization strategies. Environ Sci Technol.

[CR136] Ort C, Lawrence MG, Rieckermann J, Joss A (2010). Sampling for pharmaceuticals and personal care products (PPCPs) and illicit drugs in wastewater systems: are your conclusions valid? A critical review. Environ Sci Technol.

[CR137] Ortiz de García S, Pinto Pinto G, García Encina P, Irusta Mata R (2013). Consumption and occurrence of pharmaceutical and personal care products in the aquatic environment in Spain. Sci Total Environ.

[CR138] Pan Y, Cheng S, Yang X, Ren J, Fang J, Shang C, Song W, Lian L, Zhang X (2017). UV/chlorine treatment of carbamazepine: transformation products and their formation kinetics. Water Res.

[CR139] Paucar NE, Kim I, Tanaka H, Sato C (2019). Ozone treatment process for the removal of pharmaceuticals and personal care products in wastewater. Ozone Sci Eng.

[CR140] Peng FJ, Pan CG, Zhang M, Zhang NS, Windfeld R, Salvito D, Selck H, Van den Brink PJ, Ying GG (2017). Suppl. Occurrence and ecological risk assessment of emerging organic chemicals in urban rivers: Guangzhou as a case study in China. Sci Total Environ.

[CR141] Pérez de la Lastra JM, Anand U, Gonzále-Acosta S, López MR, Dey A, Bontempi E, Morales delaNuez A (2022). Antimicrobial resistance in the COVID-19 landscape: is there an opportunity for anti-infective antibodies and antimicrobial peptides?. Front Immunol.

[CR142] Petrie B, McAdam EJ, Lester JN, Cartmell E (2014). Obtaining process mass balances of pharmaceuticals and triclosan to determine their fate during wastewater treatment. Sci Total Environ.

[CR143] Petrie B, Barden R, Kasprzyk-Hordern B (2015). A review on emerging contaminants in wastewaters and the environment: Current knowledge, understudied areas and recommendations for future monitoring. Water Res.

[CR144] Phong Vo HN, Le GK, Hong Nguyen TM, Bui XT, Nguyen KH, Rene ER, Vo TDH, Thanh Cao ND, Mohan R (2019). Acetaminophen micropollutant: historical and current occurrences, toxicity, removal strategies and transformation pathways in different environments. Chemosphere.

[CR145] Plósz BG, Leknes H, Liltved H, Thomas KV (2010). Diurnal variations in the occurrence and the fate of hormones and antibiotics in activated sludge wastewater treatment in Oslo, Norway. Sci Total Environ.

[CR146] Poynton HC, Robinson WE, Torok B, Dransfield T (2018). Contaminants of emerging concern, with an emphasis on nanomaterials and pharmaceuticals. Green chemistry: an inclusive approach.

[CR147] Prabhasankar VP, Joshua DI, Balakrishna K, Siddiqui IF, Taniyasu S, Yamashita N, Kannan K, Akiba M, Praveenkumarreddy Y, Guruge KS (2016). Removal rates of antibiotics in four sewage treatment plants in South India. Environ Sci Pollut Res.

[CR148] Ramos S, Homem V, Santos L (2019). Simultaneous determination of synthetic musks and UV-filters in water matrices by dispersive liquid-liquid microextraction followed by gas chromatography tandem mass-spectrometry. J Chromatogr A.

[CR149] Reddersen K, Heberer T, Dünnbier U (2002). Identification and significance of phenazone drugs and their metabolites in ground- and drinking water. Chemosphere.

[CR150] Rees GM, Reyher KK, Barrett DC, Buller H (2021). ‘It’s cheaper than a dead cow’: understanding veterinary medicine use on dairy farms. J Rural Stud.

[CR151] Reyes NJDG, Geronimo FKF, Yano KAV, Guerra HB, Kim L (2021). Pharmaceutical and personal care products in different matrices: occurrence, pathways, and treatment processes. Water.

[CR152] Rice SL, Mitra S (2007). Microwave-assisted solvent extraction of solid matrices and subsequent detection of pharmaceuticals and personal care products (PPCPs) using gas chromatography-mass spectrometry. Anal Chim Acta.

[CR153] Richmond EK, Rosi EJ, Walters DM, Fick J, Hamilton SK, Brodin T, Sundelin A, Grace MR (2018). A diverse suite of pharmaceuticals contaminates stream and riparian food webs. Nat Commun.

[CR154] Roberts J, Kumar A, Du J, Hepplewhite C, Ellis DJ, Christy AG, Beavis SG (2016). Pharmaceuticals and personal care products (PPCPs) in Australia’s largest inland sewage treatment plant, and its contribution to a major Australian river during high and low flow. Sci Total Environ.

[CR155] Rodriguez-Narvaez OM, Peralta-Hernandez JM, Goonetilleke A, Bandala ER (2017). Treatment technologies for emerging contaminants in water: a review. Chem Eng J.

[CR156] Rodríguez-Narvaez OM, Rajapaksha RD, Ranasinghe MI, Bai X, Peralta-Hernández JM, Bandala ER (2020). Peroxymonosulfate decomposition by homogeneous and heterogeneous Co: kinetics and application for the degradation of acetaminophen. J Environ Sci (china).

[CR157] Rodriguez-Rodriguez CE, Jelić A, Llorca M, Farré M, Caminal G, Petrović M, Barceló D, Vicent T (2011). Solid-phase treatment with the fungus *Trametes versicolor* substantially reduces pharmaceutical concentrations and toxicity from sewage sludge. Bioresour Technol.

[CR158] Rosal R, Rodríguez A, Perdigón-Melón JA, Petre A, García-Calvo E, Gómez MJ, Agüera A, Fernández-Alba AR (2010). Occurrence of emerging pollutants in urban wastewater and their removal through biological treatment followed by ozonation. Water Res.

[CR159] Sabourin L, Duenk P, Bonte-Gelok S, Payne M, Lapen DR, Topp E (2012). Uptake of pharmaceuticals, hormones and parabens into vegetables grown in soil fertilized with municipal biosolids. Sci Total Environ.

[CR160] Saka C (2020). Analytical methods on determination in pharmaceuticals and biological materials of chloroquine as available for the treatment of COVID-19. Crit Rev Anal Chem.

[CR161] Sangion A, Gramatica P (2016). Ecotoxicity interspecies QAAR models from Daphnia toxicity of pharmaceuticals and personal care products. SAR QSAR Environ Res.

[CR162] Santana-Viera S, Guedes-Alonso R, Sosa-Ferrera Z, Santana-Rodríguez JJ, Kabir A, Furton KG (2017). Optimization and application of fabric phase sorptive extraction coupled to ultra-high-performance liquid chromatography tandem mass spectrometry for the determination of cytostatic drug residues in environmental waters. J Chromatogr A.

[CR163] Santos JL, Aparicio I, Callejón M, Alonso E (2009). Occurrence of pharmaceutically active compounds during 1-year period in wastewaters from four wastewater treatment plants in Seville (Spain). J Hazard Mater.

[CR164] Schmitt-Jansen M, Bartels P, Adler N, Altenburger R (2007). Phytotoxicity assessment of diclofenac and its phototransformation products. Anal Bioanal Chem.

[CR165] Scott PD, Bartkow M, Blockwell SJ, Coleman HM, Khan SJ, Lim R, McDonald JA, Nice H, Nugegoda D, Pettigrove V, Tremblay LA, Warne MStJ, Leusch FDL (2014) A national survey of trace organic contaminants in Australian Rivers. J Environ Qual 43(5):1702–1712. 10.2134/jeq2014.01.0012 10.2134/jeq2014.01.0012 25603256

[CR166] Senta I, Kostanjevecki P, Krizman-Matasic I, Terzic S, Ahel M (2019). Occurrence and behavior of macrolide antibiotics in municipal wastewater treatment: possible importance of metabolites, synthesis byproducts, and transformation products. Environ Sci Technol.

[CR167] Setznagl S, Cesarino I (2020). Copper nanoparticles and reduced graphene oxide modified a glassy carbon electrode for the determination of glyphosate in water samples. Int J Environ Anal Chem.

[CR168] Shalini K, Anwer Z, Sharma PK, Garg VK, Kumar N (2010). A review on pharma pollution. Int J PharmTech Res.

[CR169] Sharma BM, Bečanová J, Scheringer M, Sharma A, Bharat GK, Whitehead PG, Klánová J, Nizzetto L (2019). Health and ecological risk assessment of emerging contaminants (pharmaceuticals, personal care products, and artificial sweeteners) in surface and groundwater (drinking water) in the Ganges River Basin, India. Sci Total Environ.

[CR170] Singh V, Suthar S (2021). Occurrence, seasonal variations, and ecological risk of pharmaceuticals and personal care products in River Ganges at two holy cities of India. Chemosphere.

[CR171] Singh A, Sawant M, Kamble SJ, Herlekar M, Starkl M, Aymerich E, Kazmi A (2019). Performance evaluation of a decentralized wastewater treatment system in India. Environ Sci Pollut Res.

[CR172] Snyder SA, Adham S, Redding AM, Cannon FS, DeCarolis J, Oppenheimer J, Wert EC, Yoon Y (2007). Role of membranes and activated carbon in the removal of endocrine disruptors and pharmaceuticals. Desalination.

[CR173] Stackelberg PE, Gibs J, Furlong ET, Meyer MT, Zaugg SD, Lippincott RL (2007). Efficiency of conventional drinking-water-treatment processes in removal of pharmaceuticals and other organic compounds. Sci Total Environ.

[CR174] Stanley LA, Badal S, Delgoda R (2017). Drug metabolism. Pharmacognosy.

[CR175] Subedi B, Balakrishna K, Sinha RK, Yamashita N, Balasubramanian VG, Kannan K (2015). Mass loading and removal of pharmaceuticals and personal care products, including psychoactive and illicit drugs and artificial sweeteners, in five sewage treatment plants in India. J Environ Chem Eng.

[CR176] Sui Q, Cao X, Lu S, Zhao W, Qiu Z, Yu G (2015). Occurrence, sources and fate of pharmaceuticals and personal care products in the groundwater: a review. Emerg Contam.

[CR177] Takeuchi H, Tanaka H (2020). Water reuse and recycling in Japan—history, current situation, and future perspectives. Water Cycle.

[CR178] Tiamiyu AO, Adelodun B, Bakare HO, Ajibade FO, Kareem KY, Ibrahim RG, Odey G, Goala M, Adeniran JA, Hussain CM, Shukla SK (2021). Role of nanotechnology in coronavirus detection. Detection and analysis of SARS coronavirus.

[CR179] Trujillo-Rodríguez MJ, Nan H, Anderson JL (2018). Expanding the use of polymeric ionic liquids in headspace solid-phase microextraction: determination of ultraviolet filters in water samples. J Chromatogr A.

[CR180] US FDA (2020). Fact sheet: FDA at a glance.

[CR181] Vallecillos L, Pocurull E, Borrull F (2013). A simple and automated method to determine macrocyclic musk fragrances in sewage sludge samples by headspace solid-phase microextraction and gas chromatography-mass spectrometry. J Chromatogr A.

[CR182] Van Boeckel TP, Brower C, Gilbert M, Grenfell BT, Levin SA, Robinson TP, Teillant A, Laxminarayan R (2015). Global trends in antimicrobial use in food animals. Proc Natl Acad Sci USA.

[CR183] Vasquez MI, Lambrianides A, Schneider M, Kümmerer K, Fatta-Kassinos D (2014). Environmental side effects of pharmaceutical cocktails: what we know and what we should know. J Hazard Mater.

[CR184] Vega-Morales T, Sosa-Ferrera Z, Santana-Rodríguez JJ (2010). Determination of alkylphenol polyethoxylates, bisphenol-A, 17α-ethylestradiol and 17β-estradiol and its metabolites in sewage samples by SPE and LC/MS/MS. J Hazard Mater.

[CR185] Vellinga A, Cormican S, Driscoll J, Furey M, O’Sullivan M, Cormican M (2014). Public practice regarding disposal of unused medicines in Ireland. Sci Total Environ.

[CR186] Verlicchi P, Al Aukidy M, Galletti A, Petrovic M, Barceló D (2012). Hospital effluent: investigation of the concentrations and distribution of pharmaceuticals and environmental risk assessment. Sci Total Environ.

[CR187] Vieno N, Sillanpää M (2014). Fate of diclofenac in municipal wastewater treatment plant—a review. Environ Int.

[CR188] Vieno NM, Härkki H, Tuhkanen T, Kronberg L (2007). Occurrence of pharmaceuticals in river water and their elimination in a pilot-scale drinking water treatment plant. Environ Sci Technol.

[CR189] Vymazal J, Dvořáková Březinová T, Koželuh M, Kule L (2017). Occurrence and removal of pharmaceuticals in four full-scale constructed wetlands in the Czech Republic—the first year of monitoring. Ecol Eng.

[CR190] Wang J, Chen X (2020). Removal of antibiotic resistance genes (ARGs) in various wastewater treatment processes: an overview. Crit Rev Environ Sci Technol.

[CR191] Wang J, Chen X (2020). Catalytic ozonation for water and wastewater treatment: recent advances and perspective. Sci Total Environ.

[CR192] Wang J, Chu L (2016). Irradiation treatment of pharmaceutical and personal care products (PPCPs) in water and wastewater: an overview. Radiat Phys Chem.

[CR193] Wang J, Wang S (2016). Removal of pharmaceuticals and personal care products (PPCPs) from wastewater: a review. J Environ Manag.

[CR194] Wang J, Wang S (2017). Carbamazepine degradation by gamma irradiation coupled to biological treatment. J Hazard Mater.

[CR195] Wang J, Wang S (2021). Toxicity changes of wastewater during various advanced oxidation processes treatment: an overview. J Clean Prod.

[CR196] Wang J, Zhuan R (2020). Degradation of antibiotics by advanced oxidation processes: an overview. Sci Total Environ.

[CR197] Wang X, Yang H, Zhou B, Wang X, Xie Y (2015). Effect of oxidation on amine-based pharmaceutical degradation and N-Nitrosodimethylamine formation. Water Res.

[CR198] Wang J, Hu Y, Wang J (2018). Biodegradation of typical pharmaceutical compounds by a novel strain Acinetobacter sp. J Environ Manage.

[CR199] Wang Y, Li Y, Hu A, Rashid A, Ashfaq M, Wang Y, Wang H, Luo H, Yu CP, Sun Q (2018). Monitoring, mass balance and fate of pharmaceuticals and personal care products in seven wastewater treatment plants in Xiamen City, China. J Hazard Mater.

[CR200] Wang J, Zhuan R, Chu L (2019). The occurrence, distribution and degradation of antibiotics by ionizing radiation: an overview. Sci Total Environ.

[CR201] Wang J, Chu L, Wojnarovits L, Takacs E (2020). Occurrence and fate of antibiotics, antibiotic resistant genes (ARGs) and antibiotic resistant bacteria (ARB) in municipal wastewater treatment plant: an overview. Sci Total Environ.

[CR202] WHO (2003). WHO policy perspectives on medicines—how to develop and implement a national drug policy.

[CR203] WHO (2012). Pharmaceuticals in drinking water.

[CR204] Williams RJ, Johnson AC, Smith JJL, Kanda R (2003). Steroid estrogens profiles along river stretches arising from sewage treatment works discharges. Environ Sci Technol.

[CR205] WWAP (2017) (United Nations World Water Assessment Programme). The United Nations World Water Development Report 2017. Wastewater: the untapped resource. Paris, UNESCO, vol 53, issue 9

[CR206] Xiang Y, Wu H, Li L, Ren M, Qie H, Lin A (2021). A review of distribution and risk of pharmaceuticals and personal care products in the aquatic environment in China. Ecotoxicol Environ Saf.

[CR207] Xiao J, Xie Y, Cao H (2015). Organic pollutants removal in wastewater by heterogeneous photocatalytic ozonation. Chemosphere.

[CR208] Yang X, Chen F, Meng F, Xie Y, Chen H, Young K, Luo W, Ye T, Fu W (2013). Occurrence and fate of PPCPs and correlations with water quality parameters in urban riverine waters of the Pearl River Delta, South China. Environ Sci Pollut Res.

[CR209] Yang Y, Ok YS, Kim K-H, Kwon EE, Tsang YF (2017). Occurrences and removal of pharmaceuticals and personal care products (PPCPs) in drinking water and water/sewage treatment plants: a review. Sci Total Environ.

[CR210] Yang H, Lu G, Yan Z, Liu J, Dong H, Bao X, Zhang X, Sun Y (2020). Residues, bioaccumulation, and trophic transfer of pharmaceuticals and personal care products in highly urbanized rivers affected by water diversion. J Hazard Mater.

[CR211] Yeh A, Marcinek DJ, Meador JP, Gallagher EP (2017). Effect of contaminants of emerging concern on liver mitochondrial function in Chinook salmon. Aquat Toxicol.

[CR212] Yin L, Wang B, Yuan H, Deng S, Huang J, Wang Y, Yu G (2017). Pay special attention to the transformation products of PPCPs in environment. Emerg Contam.

[CR213] Zaidi STR, Hasan SS (2021). Personal protective practices and pharmacy services delivery by community pharmacists during COVID-19 pandemic: results from a national survey. Res Social Adm Pharm.

[CR214] Zhan Y, Hong N, Yang B, Du Y, Wu Q, Liu A (2020). Toxicity variability of urban road stormwater during storage processes in Shenzhen, China: identification of primary toxicity contributors and implications for reuse safety. Sci Total Environ.

[CR215] Zhang X, Zhao H, Du J, Qu Y, Shen C, Tan F, Chen J, Quan X (2017). Occurrence, removal, and risk assessment of antibiotics in 12 wastewater treatment plants from Dalian, China. Environ Sci Pollut Res.

[CR216] Zhang Z, Chen H, Wang J, Zhang Y (2020). Degradation of carbamazepine by combined radiation and persulfate oxidation process. Radiat Phys Chem.

[CR217] Zhang T, Xu Q, Shi Y, Chen Z, Lu Y, Yang H, Xie YF, Hou L (2021). Study on the influence of operational and management processes of a water reclamation plant since COVID-19 situation. Environ Pollut.

[CR218] Zuccato E, Castiglioni S, Fanelli R (2005). Identification of the pharmaceuticals for human use contaminating the Italian aquatic environment. J Hazard Mater.

[CR219] Zühlke S, Dünnbier U, Heberer T (2004). Detection and identification of phenazone-type drugs and their microbial metabolites in ground and drinking water applying solid-phase extraction and gas chromatography with mass spectrometric detection. J Chromatogr A.

