# Supplementary material for: Occurrence, transformation, bioaccumulation, risk and analysis of pharmaceutical and personal care products from wastewater: a review
Source: Environ Chem Lett. 2022 Aug 17;20(6):3883–904. doi: 10.1007/s10311-022-01498-7 (PMC9385088; doi:10.1007/s10311-022-01498-7)
Supplement: Supplementary file 1 — (DOCX 2062 kb) [file 10311_2022_1498_MOESM1_ESM.docx]

**Figure** **S1.** Numbers of research papers appearing with “Impact of pharmaceuticals, personal care products, wastewater and COVID-19”, in the topic as listed in the ScienceDirect, NCBI-PMC (US National Library of Medicine National Institutes of Health) and Google Scholar databases (database searched December 03, 2021) . In the Google Scholar 342 articles are appearing in 2019 and other two search engines showed null. This figure clearly shows an increased interest in pharmaceutical and personal care products and COVID-19-related studies in water and wastewater compartments.


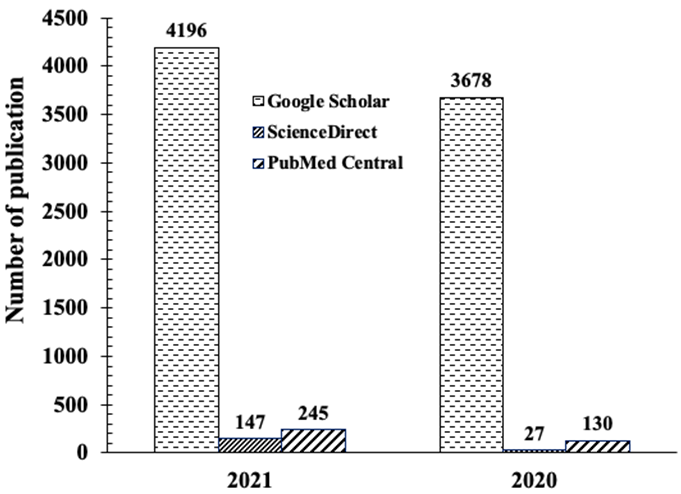


**Figure S2.** Flowchart of the literature search and selection criteria, updated on December 15, 2021, that allowed identifying the most suitable articles for the present work. To select a restricted and suitable number of papers, a specific search procedure was performed, by using the SCOPUS database (<https://www.scopus.com/>). The first exploratory search involved the search of ("emerging contaminants”) OR (“emerging pollutants”) in the papers title, abstract, and keywords, to select the most relevant studies. Then, due to a high number of available papers (6740), the initial sample was restricted only to keywords. Keyword search is a suitable way to highlight articles that are relevant only for study purposes. The following selection strategy involved the inclusion in all search fields of the “wastewater” and “health” words. The combined search allowed us to identify a total of 2168
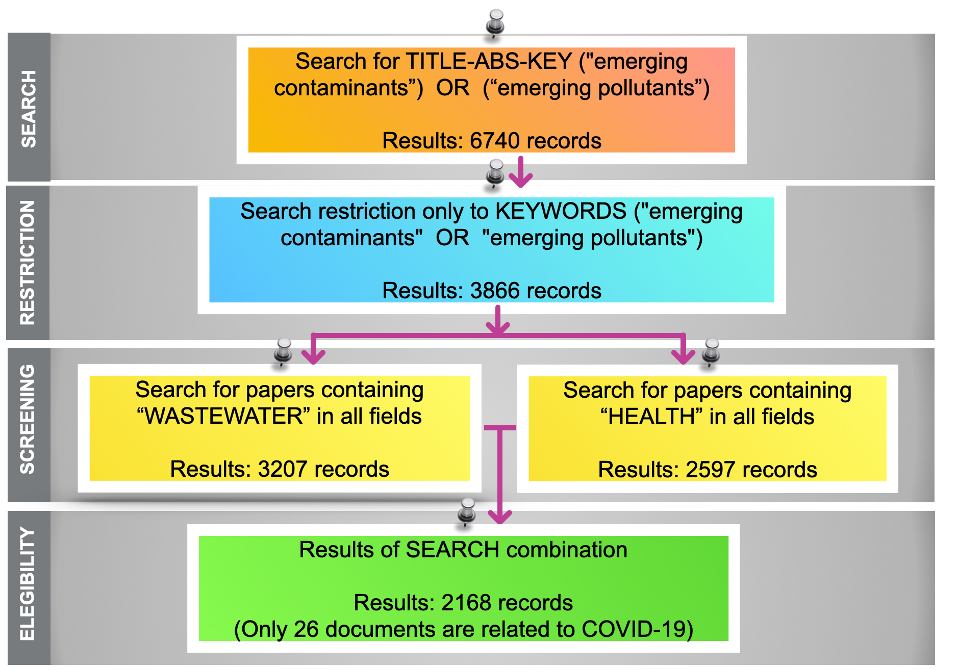
articles. Only 26 documents concerned COVID-19.

**Figure S3.** Map of the frequently used terms co-occurrence network made on the selected bibliography (for the search criteria see **Figure S2**). The map (realised with VOSviewer https://www.vosviewer.com) corresponds to a 2D representation of a research field, in which it is possible to highlight strongly related terms, that result located in proximity. This procedure allows focalising the prevailing thematic flow. The analysis resulted in a total of 782 terms grouped in 5 clusters. The frequently used terms co-occurrence network is divided into 5 clusters, with different colours (cluster 1: red, cluster 2: green, cluster 3: blue, cluster 4: yellow, cluster 5: purple). The bubbles with larger diameters are “occurrence” and “removal”, corresponding to most of the output related to the selected bibliography. The red cluster (number 1) is related to the pharmaceutical and personal care products (PPCPs) occurrence and environmental distribution. It groups the higher number of recurring terms (351 items). Clusters 2 and 3 (green and blue respectively) are devoted to PPCPs removal and degradation with attention to toxicological characteristics. Another research trend corresponds to the PPCP detection, determination, and extraction methods (yellow bubbles, corresponding to cluster 4). The purple circles (cluster 5) are related to the human health risk
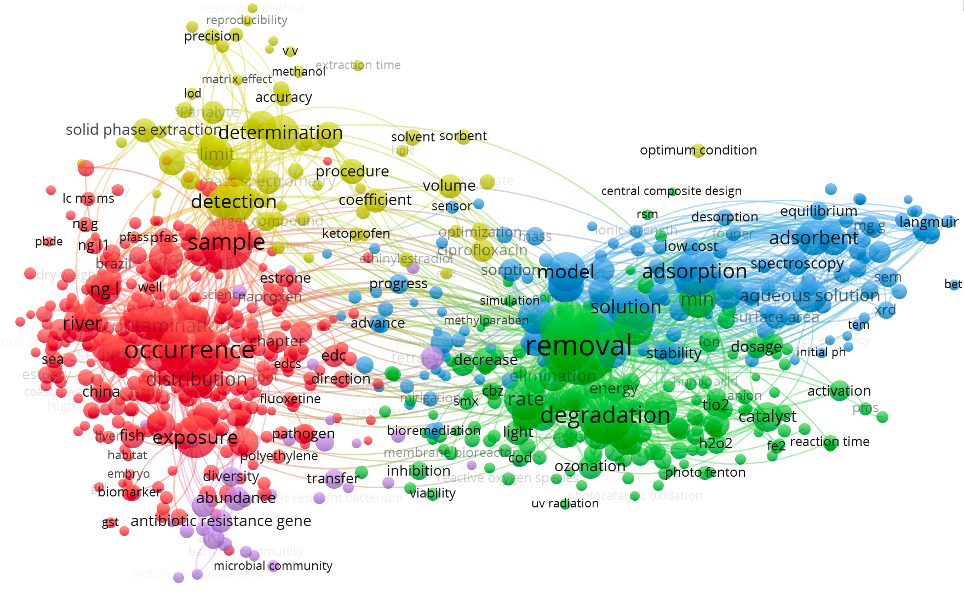
assessment of PPCPs.

**Table S4.** List of some pharmaceutical and personal care products compounds detected in WWTP effluents and adjacent water bodies.

| **Name** | **Abbreviation** | **Use type** | **Reference** |
| --- | --- | --- | --- |
| 17β-estradiol | βE2 | Natural hormone | (Nazari and Suja, 2016; Williams et al., 2003) |
| 17α-Ethinylestradiol | EE2 | Contraceptive | (Williams et al., 2003) |
| Acetaminophen (Paracetamol) | ATP | Antipyretic analgesic | (Ashfaq et al., 2017; Kosma et al., 2014; Kumar et al., 2019; Lin et al., 2018; Wang et al., 2018) |
| Acetophenone | APN | Fragrance, food additive | (Ashfaq et al., 2017; Wang et al., 2018) |
| Acesulfame | ASF | Artificial sweetener | (Subedi et al., 2015) |
| Alprazolam | APZ | Anxiolytic | (Subedi et al., 2015) |
| Amphetamine | APT | Illicit drug | (Subedi et al., 2015) |
| Antipyrine | ANT | Antipyretic analgesic | (Ashfaq et al., 2017; Lin et al., 2018) |
| Aripiprazole | APPZ | Antischizophrenic | (Subedi et al., 2015) |
| Aspartame | AST | Artificial sweetener | (Ashfaq et al., 2017; Wang et al., 2018) |
| Atenolol (Tenormin) | ANL | Antihypertension | (Kumar et al., 2019; Subedi et al., 2015) |
| Atrazine | ATZ | Herbicide | (Kumar et al., 2019) |
| Azithromycin | AZT | Antibiotic | (Lin et al., 2018) |
| Benzotriazole | BTA | Corrosion inhibitor | (Kumar et al., 2019) |
| Benzoylecgonine | BEG | Illicit drug | (Subedi et al., 2015) |
| Bezafibrate | BZF | Blood lipid regulator | (Dai et al., 2014; Kosma et al., 2014) |
| Bisphenol-A | BPA | Plasticizer | (Kumar et al., 2019) |
| Bupropion hydrochloride | BPP | Antidepressant | (Subedi et al., 2015) |
| Budesonide | BUD | Anti-inflammatory | (Kosma et al., 2014) |
| Caffeine | CAF | Stimulant | (Ashfaq et al., 2017; Dai et al., 2014; Kosma et al., 2014; Kumar et al., 2019; Subedi et al., 2015; Wang et al., 2018) |
| Carbamazepine | CBZ | Anticonvulsant | (Ashfaq et al., 2017; Dai et al., 2014; Franklin et al., 2016; Kosma et al., 2014; Kumar et al., 2019; Subedi et al., 2015; Wang et al., 2018) |
| Chloramphenicol | CAP | Antibiotic | (Dai et al., 2014; Lin et al., 2018) |
| Chlortetracycline | CTC | Antibiotic | (Ashfaq et al., 2017; Lin et al., 2018) |
| Ciprofloxacin | CIP | Antibiotic | (Ashfaq et al., 2017; Lin et al., 2018; Wang et al., 2018) |
| Clarithromycin | CAM | Antibiotic | (Kumar et al., 2019; Lin et al., 2018) |
| Clindamycin | CDM | Antibiotic | (Subedi et al., 2015) |
| Clofibric acid | CA | Blood lipid regulator | (Ashfaq et al., 2017; Dai et al., 2014; Kosma et al., 2014; Wang et al., 2018) |
| Cocaine | CCN | Illicit drug | (Subedi et al., 2015) |
| Codeine | CDN | Analgesic | (Subedi et al., 2015) |
| Cyclamate | CCM | Artificial sweetener | (Subedi et al., 2015) |
| Cyclophosphamide | CPM | Antineoplastic | (Ashfaq et al., 2017; Wang et al., 2018) |
| Danofloxacin | DFX | Antibiotic | (Ashfaq et al., 2017) |
| Desacetyl diltiazem | DAD | Antihypertension | (Subedi et al., 2015) |
| Dexamethasone | DEX | Anti-inflammatory | (Desgens-Martin and Keller, 2021) |
| Diazepam | DIA | Anxiolytic | (Ashfaq et al., 2017; Subedi et al., 2015; Wang et al., 2018) |
| Diclofenac | DIC | Anti-inflammatory | (Ashfaq et al., 2017; Dai et al., 2014; Kosma et al., 2014; Kumar et al., 2019; Lin et al., 2018) |
| Difloxacin | DIF | Antibiotic | (Lin et al., 2018) |
| Diltiazem hydrochloride |  | Antihypertension | (Subedi et al., 2015) |
| Diphenhydramine | DIP | Antihistamine | (Subedi et al., 2015) |
| Doxycycline | DOX | Antibiotic | (Lin et al., 2018) |
| Enrofloxacin | ENR | Antibiotic | (Ashfaq et al., 2017; Lin et al., 2018; Wang et al., 2018) |
| Erythromycin | ERY | Antibiotic | (Lin et al., 2018) |
| Fenofibrate | FNB | Blood lipid regulator | (Kosma et al., 2014) |
| Fenoprofen | FPF | Anti-inflammatory | (Ashfaq et al., 2017; Wang et al., 2018) |
| Florfenicol | FF | Antibiotic | (Lin et al., 2018) |
| Fluoxetine | FLX | Antidepressant | (Ashfaq et al., 2017; Kumar et al., 2019; Wang et al., 2018) |
| Flumequine | FMQ | Antibiotic | (Lin et al., 2018) |
| Gemfibrozil | GMF | Blood lipid regulator | (Ashfaq et al., 2017; Dai et al., 2014; Kosma et al., 2014; Wang et al., 2018) |
| Glyburide (Glibenclamide) | GLY | Hypoglycemic agent | (Ashfaq et al., 2017) |
| Ibuprofen | IBU | Anti-inflammatory | (Ashfaq et al., 2017; Kosma et al., 2014; Kumar et al., 2019; Lin et al., 2018; Subedi et al., 2015; Wang et al., 2018) |
| Indomethacin | IM | Anti-inflammatory | (Ashfaq et al., 2017; Dai et al., 2014; Lin et al., 2018; Wang et al., 2018) |
| Ketoprofen | KET | Anti-inflammatory | (Ashfaq et al., 2017; Lin et al., 2018; Subedi et al., 2015; Wang et al., 2018) |
| Lincomycin | LNC | Antibiotic | (Lin et al., 2018; Subedi et al., 2015) |
| Lorazepam | LZP | Anxiolytic | (Subedi et al., 2015) |
| Losartan | LST | Antihypertension | (Ashfaq et al., 2017; Wang et al., 2018) |
| Mefenamic acid | MA | Anti-inflammatory | (Ashfaq et al., 2017; Dai et al., 2014; Lin et al., 2018; Wang et al., 2018) |
| Methamphetamine | MAA | Illicit drug | (Subedi et al., 2015) |
| Methylparaben | MP | Preservative | (Ashfaq et al., 2017; Wang et al., 2018) |
| Metoprolol | MTP | Antihypertension | (Ashfaq et al., 2017; Dai et al., 2014; Kumar et al., 2019; Wang et al., 2018) |
| Miconazole | MIC | Antifungal | (Ashfaq et al., 2017; Subedi et al., 2015; Wang et al., 2018) |
| Morphine | MPH | Illicit drug | (Subedi et al., 2015) |
| N,N-Diethyl-m-toluamide | DEET | Insecticide | (Dai et al., 2014; Kumar et al., 2019) |
| Nalidixic acid | NA | Antibiotic | (Dai et al., 2014) |
| Naproxen | NAP | Anti-inflammatory | (Ashfaq et al., 2017; Kosma et al., 2014; Kumar et al., 2019; Lin et al., 2018; Wang et al., 2018) |
| Norfloxacin | NOR | Antibiotic | (Ashfaq et al., 2017; Lin et al., 2018; Wang et al., 2018) |
| Norquetiapine hydrochloride | NQTP | Antischizophrenic | (Subedi et al., 2015) |
| Octocrylene | OCT | UV absorber | (Ashfaq et al., 2017) |
| Ofloxacin | OFL | Antibiotic | (Ashfaq et al., 2017; Franklin et al., 2016; Lin et al., 2018; Wang et al., 2018) |
| Oxazepam | OZP | Sedative | (Subedi et al., 2015) |
| Oxybenzone (Benzophenone-3) | OXB | UV-filter | (Subedi et al., 2015; Wang et al., 2018) |
| Oxytetracycline | OTC | Antibiotic | (Ashfaq et al., 2017; Lin et al., 2018; Wang et al., 2018) |
| Phenazone | PNZ | Anti-inflammatory | (Kosma et al., 2014) |
| Propyphenazone | PPNZ | Anti-inflammatory | (Ashfaq et al., 2017; Wang et al., 2018) |
| Propranolol hydrochloride | PHO | Antiarrhythmic | (Ashfaq et al., 2017; Dai et al., 2014; Subedi et al., 2015; Wang et al., 2018) |
| Propylparaben | PP | Preservative | (Ashfaq et al., 2017; Wang et al., 2018) |
| Quetiapine fumarate | QTP | Antischizophrenic | (Subedi et al., 2015) |
| Remdesivir | RDV | Antiviral | (Desgens-Martin and Keller, 2021) |
| Roxithromycin | ROX | Antibiotic | (Kumar et al., 2019; Lin et al., 2018) |
| Saccharin | SCH | Artificial sweetener | (Subedi et al., 2015) |
| Salicylic acid | SA | Antiseptic | (Kosma et al., 2014) |
| Sarafloxacin | SAR | Antibiotic | (Ashfaq et al., 2017) |
| Sertraline hydrochloride | STH | Antidepressant | (Subedi et al., 2015) |
| Sucralose | SCL | Artificial sweetener | (Subedi et al., 2015) |
| Sulfadiazine | SDZ | Antibiotic | (Ashfaq et al., 2017; Lin et al., 2018) |
| Sulfamerazine | SMR | Antibiotic | (Lin et al., 2018) |
| Sulfamethazine | SMZ | Antibiotic | (Ashfaq et al., 2017; Lin et al., 2018) |
| Sulfamethoxazole | SMX | Antibiotic | (Ashfaq et al., 2017; Franklin et al., 2016; Kosma et al., 2014; Kumar et al., 2019; Lin et al., 2018; Subedi et al., 2015) |
| Sulfaquinoxaline | SQX | Antibiotic | (Lin et al., 2018) |
| Sulfathiazole | STZ | Antibiotic | (Lin et al., 2018) |
| Sulpiride | SP | Antipsychotic | (Dai et al., 2014) |
| Tetracycline | TC | Antibiotic | (Ashfaq et al., 2017; Lin et al., 2018; Wang et al., 2018) |
| Thiabendazole | TBZ | Antihelmintic | (Ashfaq et al., 2017) |
| Thiamphenicol | THI | Antibiotic | (Lin et al., 2018) |
| Tolfenamic acid | TA | Anti-inflammatory | (Kosma et al., 2014) |
| Triclocarban | TCC | Disinfectant | (Subedi et al., 2015; Wang et al., 2018) |
| Triclosan | TCS | Disinfectant | (Kosma et al., 2014; Kumar et al., 2019; Subedi et al., 2015; Wang et al., 2018) |
| Trimethoprim | TMP | Antibiotic | (Dai et al., 2014; Franklin et al., 2016; Kosma et al., 2014; Kumar et al., 2019; Lin et al., 2018; Subedi et al., 2015) |
| Tris(2-carboxyethyl)phosphine | TCEP | Reducing agent | (Kumar et al., 2019) |
| Tylosin | TYL | Antibiotic | (Lin et al., 2018) |

**Table S5**. Instrumental techniques used for specific chemicals detection. Mass spectrometry (MS), Mass spectrometry coupled with gas-phase chromatography (GC-MS), liquid-phase chromatography (LC-MS), and High-Performance Liquid Chromatography (HPLC) (Meng et al., 2021).

| **Category** | **Compound name** | **Structural formula** | **Instrumental analysis** | **Reference** |
| --- | --- | --- | --- | --- |
| Aminoglycosides | Spectinomycin | 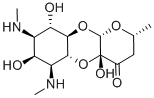 | LC-MS/MS | Li et al., 2018 |
|  | Streptomycin | 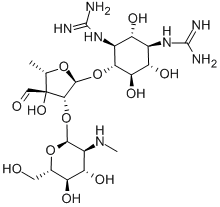 | LC-MS/MS | Li et al., 2018 |
| Amphenicols | Chloramphenicol | 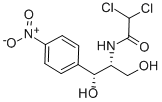 | LC-MS/MS | Li et al., 2018 |
|  | Thiamphenicol | 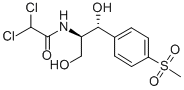 | LC-MS/MS | Li et al., 2018 |
| Quinolones | Ciprofloxacin | 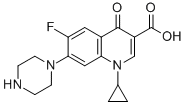 | LC-MS/MS | Li et al., 2018 |
|  | Danofloxacin mesylate | 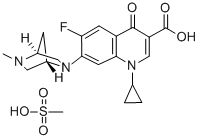 | LC-MS/MS | Wang et al., 2018 |
|  | Enrofloxacin | 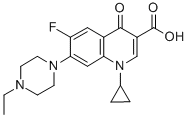 | LC-MS/MS | Wang et al., 2018 |
|  | Flumequine | 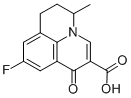 | LC-MS/MS | Li et al., 2018 |
|  | Norfloxacin | 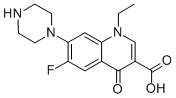 | LC-MS/MS | Li et al., 2018 |
|  | Ofloxacin | 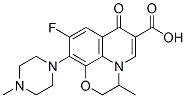 | LC-MS/MS | Li et al., 2018 |
|  | Sarafloxacin  Hydrochloride | 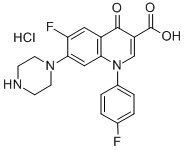 | LC-MS/MS | Wang et al., 2018 |
| Sulfonamides | Sulfadiazine | 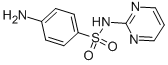 | LC-MS | Li et al., 2018 |
|  | Sulfamerazine | 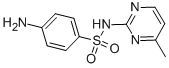 | LC-MS/MS | Li et al., 2018 |
|  | Sulfamethazine | 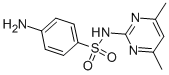 | LC-MS/MS | Li et al., 2018 |
|  | Sulfamethoxazole | 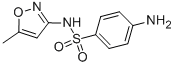 | LC-MS/MS | Li et al., 2018 |
|  | Sulfapyridine | 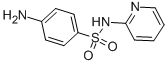 | LC-MS | Li et al., 2018 |
|  | Sulfathiazole | 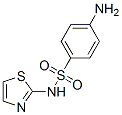 | LC-MS | Li et al., 2018 |
|  | Trimethoprim | 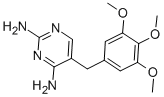 | LC-MS/MS | Li et al., 2018 |
| Tetracyclines | Isochlortetracyclinehydrochloride | 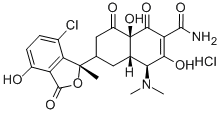 | LC-MS/MS | Li et al., 2018 |
|  | Oxytetracycline | 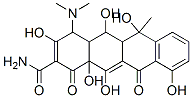 | LC-MS/MS | Li et al., 2018 |
|  | Tetracycline | 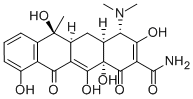 | LC-MS/MS | Li et al., 2018 |
| β-lactams | Amoxicillin | 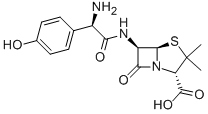 | LC-MS/MS | Li et al., 2018 |
|  | Ampicillin | 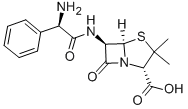 | LC-MS/MS | Li et al., 2018 |
|  | Cefotaxime | 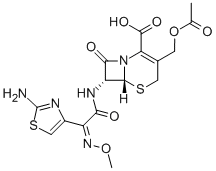 | LC-MS/MS | Li et al., 2018 |
|  | Cephalexin | 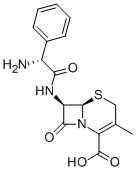 | LC-MS/MS | Li et al., 2018 |
|  | Penicillin G | 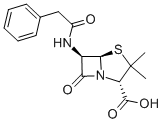 | LC-MS/MS | Li et al., 2018 |
| Macrolides | Anhydroerythromycin | 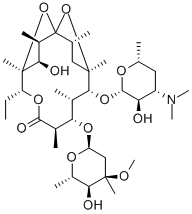 | LC-MS/MS | Li et al., 2018 |
|  | Clarithromycin | 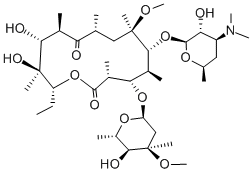 | LC-MS/MS | Caldas et  al., 2016 |
|  | Erythromycin | 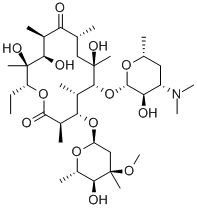 | LC-MS/MS | Li et al., 2018 |
|  | Roxithromycin | 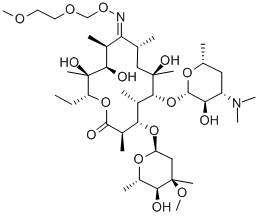 | LC-MS/MS | Li et al., 2018 |
| Cytostatic compounds | Cyclophosphamide | 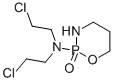 | LC-MS | Santana-Viera et al., 2017 |
|  | Etoposide | 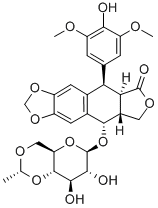 | LC-MS | Santana-Viera et al., 2017 |
|  | Gemcitabine | 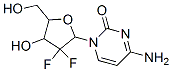 | LC-MS | Santana-Viera et al., 2017 |
|  | Methotrexate | 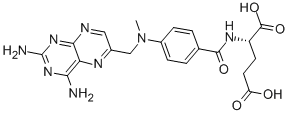 | LC-MS | Santana-Viera et al., 2017 |
|  | Tamoxifen | 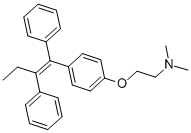 | LC-MS | Santana-Viera et al., 2017 |
|  | Vinblastine | 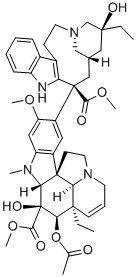 | LC-MS | Santana-Viera et al., 2017 |
|  | Vincristine | 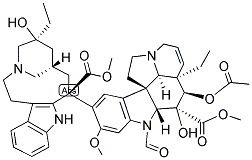 | LC-MS | Santana-Viera et al., 2017 |
| Antidiabetic | Metformin | 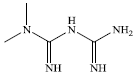 | LC-MS | Kumar et al., 2019 |
| Stimulant | Caffeine | 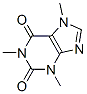 | LC–MS/MS, GC–MS | Bratkowska et al., 2011 |
| Diuretic | Furosemide | 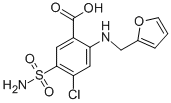 | LC-MS/MS | Caldas et al., 2016 |
|  | Hydrochlorothiazide | 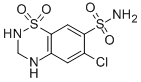 | LC-MS | Huerta et al., 2013 |
| β-blockers | Atenolol | 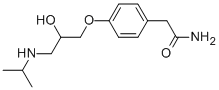 | LC-MS | Huerta et al., 2013 |
|  | Carazolol | 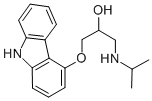 | LC-MS | Huerta et al., 2013 |
|  | Metoprolol | 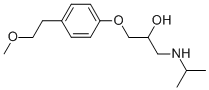 | LC-MS | Bratkowska et al., 2011 |
|  | Nadolol | 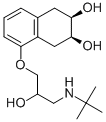 | LC-MS | Huerta et al., 2013 |
|  | Propranolol | 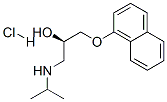 | LC-MS/MS | Caldas et al., 2016 |
|  | Sotalol | 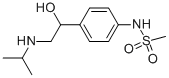 | LC-MS | Wang et al., 2018 |
| Analgesia - antipyretic drug | Antipyrine | 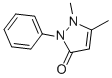 | LC–MS/MS | Bratkowska et al., 2011 |
| NSAIDs | 2-Ethoxybenzamide | 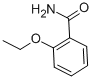 | LC-MS | Wang et al., 2018 |
|  | Acetaminophen | 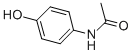 | LC-MS/MS | Wang et al., 2018 |
|  | Acetylsalicylic acid | 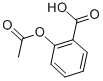 | GC-MS | Arismendi et al., 2019 |
|  | Fenoprofen | 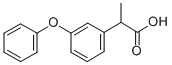 | LC-MS | Wang et al., 2018 |
|  | Ibuprofen | 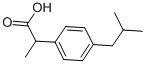 | LC-MS/MS，GC-MS | Caldas et al., 2016 |
|  | Indomethacin | 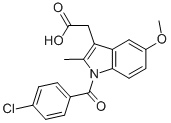 | LC-MS | Wang et al., 2018 |
|  | Ketoprofen | 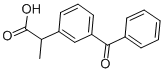 | GC-MS, LC-MS | Arismendi et al., 2019 |
|  | Mefenamic acid | 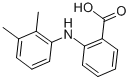 | GC-MS | Arismendi et al., 2019 |
|  | Naproxen | 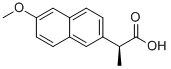 | GC-MS, LC-MS | Arismendi et al., 2019 |
|  | Nimesulide | 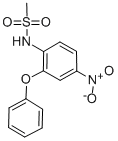 | LC-MS/MS | Bratkowska et al., 2011 |
|  | Propyphenazone | 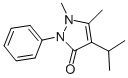 | LC-MS | Wang et al., 2018 |
|  | Salicylic acid | 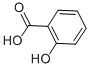 | LC-MS/MS | Caldas et al., 2016 |
|  | Sodium diclofenac | 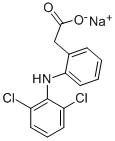 | LC-MS/MS, GC-MS | Caldas et al., 2016 |
| Antineoplastics | Cyclophosphamid | 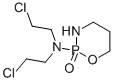 | LC-MS/MS | Wang et al., 2018 |
| Asthma treated drugs | Salbutamol | 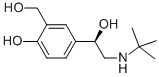 | LC-MS | Huerta et al., 2013 |
| Hypertensive agent | Losartan | 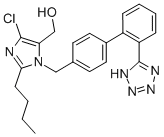 | LC-MS/MS | Wang et al., 2018 |
| Drugs of abuse | Codeine | 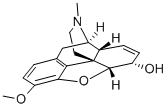 | LC-MS | Huerta et al., 2013 |
| Insect repellent | Diethyltoluamide | 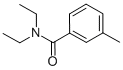 | LC-MS | Kumar et al., 2019 |
| Ulcer drug | Pirenzepine | 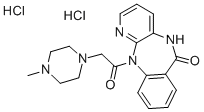 | LC-MS/MS | Wang et al., 2018 |
| Preservatives | 1H-Benzotriazole | 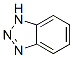 | LC-MS | Kumar et al., 2019 |
|  | Butylparaben | 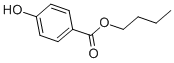 | LC-MS/MS | Lei et al., 2018 |
|  | Ethyl paraben | 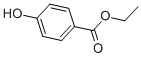 | LC-MS/MS | Lei et al., 2018 |
|  | Methylparaben | 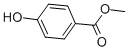 | LC-MS/MS; GC-MS | Lei et al., 2018 |
|  | Para-hydroxybenzoic acid | 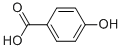 | LC-MS/MS | Lei et al., 2018 |
|  | Propylparaben | 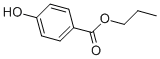 | LC-MS/MS | Lei et al., 2018 |
| Anthelminthic | 4-Hydroxyfenbendazole | 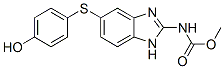 | HPLC | Kim et al., 2010 |
|  | Albendazole | 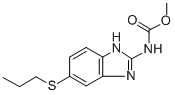 | LC-MS/MS | Caldas et al., 2016 |
|  | Fenbendazole | 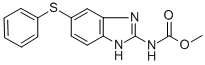 | HPLC | Kim et al., 2010 |
|  | Fenbendazole sulfone | 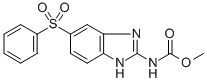 | HPLC | Kim et al., 2010 |
|  | Fenbendazoleamine | 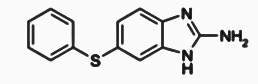 | HPLC | Kim et al., 2010 |
|  | Flubendazole | 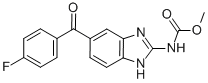 | HPLC | Kim et al., 2010 |
|  | Mebendazole | 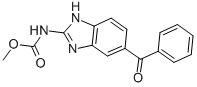 | LC-MS/MS | Caldas et al., 2016 |
|  | Oxfendazole | 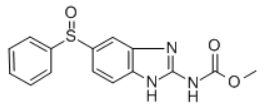 | HPLC | Kim et al., 2010 |
|  | Thiabendazole | 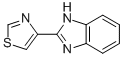 | HPLC | Kim et al., 2010 |
| β-sympathomimetic | Clenbuterol | 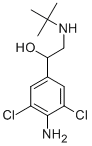 | LC-MS | Wang et al., 2018 |
| Hypoglycemic Agent | Chlorpropamide | 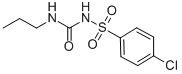 | LC-MS/MS | Caldas et al., 2016 |
|  | Glibenclamide | 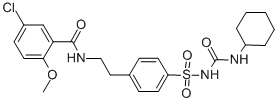 | LC-MS/MS | Caldas et al., 2016 |
| Biological response modifier | Levamisole | 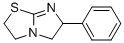 | LC-MS | Huerta et al., 2013 |
| Psychiatric drugs | Carbamazepine | 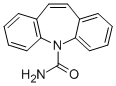 | LC-MS/MS | Caldas et al., 2016 |
|  | Citalopram | 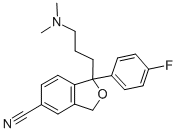 | LC-MS | Huerta et al., 2013 |
|  | Diazepam | 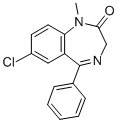 | LC-MS/MS | Huerta et al., 2013 |
|  | Haloperidol | 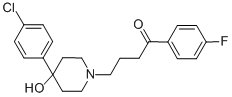 | LC-MS/MS | Caldas et al., 2016 |
|  | Lorazepam | 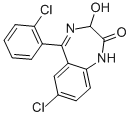 | LC-MS | Huerta et al., 2013 |
|  | Sertraline | 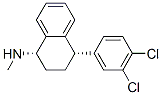 | LC-MS | Huerta et al., 2013 |
|  | Venlafaxine | 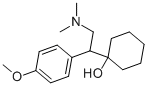 | LC-MS | Huerta et al., 2013 |
| Antipruritic | Crotamiton | 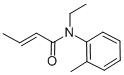 | LC-MS/MS | Wang et al., 2018 |
| Calcium channel blockers | Diltiazem hydrochloride | 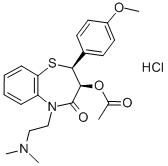 | LC-MS/MS | Caldas et al., 2016 |
| Lipid regulator | Gemfibrozil | 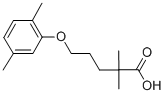 | LC-MS/MS | Caldas et al., 2016 |
| Antidepressants | Amitriptyline | 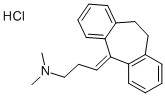 | LC-MS/MS | Caldas et al., 2016 |
|  | Fluoxetine |  | LC-MS/MS | Wang et al., 2018 |
|  | Flurazepam hydrochloride |  | LC-MS/MS | Caldas et al., 2016 |
| Central nervous system depressants | Diphenhydramine Hydrochloride |  | GC–MS | Rice and Mitra, 2007 |
|  | Pridinol |  | LC-MS/MS | Gilart et al., 2013 |
| Standardized Chemical Allergen | Benzylparaben |  | LC-MS/MS | Gilart et al., 2013 |
| Endocrine-disrupting chemicals | 17-α-Ethinylestradiol |  | GC-MS; LC-MS/MS | Arismendi et al., 2019 |
|  | 17-β-estradiol |  | GC-MS; LC-MS/MS | Arismendi et al., 2019 |
|  | 17-β- Estriol |  | GC-MS; LC-MS/MS | Arismendi et al., 2019 |
|  | Bisphenol-A |  | LC-MS/MS | Vega-Morales et al., 2010 |
|  | Estrone |  | GC-MS; LC-MS/MS | Arismendi et al., 2019 |
| Disinfectants | Miconazole nitrate |  | LC-MS/MS | Caldas et al., 2016 |
|  | Ortho-phenylphenol |  | LC-MS/MS | Lei et al., 2018 |
|  | Triclocarban |  | LC-MS/MS | Caldas et al., 2016 |
|  | Triclosan |  | LC-MS/MS，GC-MS | Caldas et al., 2016 |
| Antiplatelet agent | Clopidogrel |  | LC-MS | Huerta et al., 2013 |
| UV Filters | Ambrettolide |  | GC–MS | Vallecillos et al., 2013 |
|  | Cashmeran |  | GC–MS/MS | Ramos et al., 2019 |
|  | Celestolide |  | GC–MS/MS | Ramos et al., 2019 |
|  | Civetone |  | GC–MS | Vallecillos et al., 2013 |
|  | 2-Ethylhexyl 4-dimethylaminobenzoate |  | GC–MS/MS | Ramos et al., 2019 |
|  | 2-ethylhexyl 4-methoxycinnamate |  | GC–MS | Trujillo-Rodríguez et al., 2018 |
|  | 2-ethylhexyl salicylate |  | GC–MS | Trujillo-Rodríguez et al., 2018 |
|  | 2,4-dihydroxy-benzophenone |  | LC–MS/MS | Bratkowska et al., 2011 |
|  | 3-(4’- methylbenzylidene) camphor |  | GC–MS/MS | Ramos et al., 2019 |
|  | 4-hydroxybenzophenone |  | GC-MS | Kotnik et al., 2014 |
|  | Avobenzone |  | LC-MS/MS | Caldas et al., 2016 |
|  | Benzophenone |  | GC–MS/MS | Ramos et al., 2019 |
|  | Benzyl-salicylate |  | GC–MS | Trujillo-Rodríguez et al., 2018 |
|  | Drometrizole trisiloxane |  | GC–MS/MS | Ramos et al., 2019 |
|  | Enzophenone-8 |  | LC–MS/MS | Bratkowska et al., 2011 |
|  | Ethylhexyl dimethyl PABA |  | GC–MS | Trujillo-Rodríguez et al., 2018 |
|  | Etocrylen |  | GC–MS | Trujillo-Rodríguez et al., 2018 |
|  | Homosalate |  | GC–MS | Trujillo-Rodríguez et al., 2018 |
|  | Methyl  anthranilate |  | GC–MS | Trujillo-Rodríguez et al., 2018 |
|  | Octinoxate |  | GC-MS | Basaglia et al., 2012 |
|  | Octocrylene |  | GC–MS/MS | Ramos et al., 2019 |
|  | Oxybenzone |  | LC–MS/MS | Bratkowska et al., 2011 |
| Fragrances | Ethylene brassylate |  | GC–MS/MS | Vallecillos et al., 2013 |
|  | Ethylene dodecanedioate |  | GC–MS | Vallecillos et al., 2013 |
|  | Exaltolide |  | GC–MS/MS | Ramos et al., 2019 |
|  | Galaxolide |  | GC–MS/MS | Ramos et al., 2019 |
|  | Habanolide |  | GC–MS | Vallecillos et al., 2013 |
|  | Muscone |  | GC–MS | Vallecillos et al., 2013 |
|  | Musk ambrette |  | GC–MS/MS | Ramos et al., 2019 |
|  | Musk Ketone |  | GC–MS | Rice and Mitra, 2007 |
|  | Musk moskene |  | GC–MS/MS | Ramos et al., 2019 |
|  | Musk tibetene |  | GC–MS/MS | Ramos et al., 2019 |
|  | Musk xylene |  | GC–MS/MS | Ramos et al., 2019 |
|  | Phantolide |  | GC–MS/MS | Ramos et al., 2019 |
|  | Tonalide |  | GC–MS/MS | Ramos et al., 2019 |
|  | Traseolide |  | GC–MS/MS | Ramos et al., 2019 |
| Antioxidants | Butylated hydroxyanisole |  | LC-MS/MS | Lei et al., 2018 |
|  | Butylated hydroxytoluene |  | GC/MS | Basaglia et al., 2012 |

**Table S6.** Presence of pharmaceutical and personal care products in different environmental samples along with treatment processes in selected countries.

| **Classification** | **Compounds of pharmaceutical and personal care products** | **Media/**  **sample** | **Location**  **source or pathway** | **Concentration (unit)** | | **Decontamination/**  **treatment** | **References** |
| --- | --- | --- | --- | --- | --- | --- | --- |
|  |  |  |  | **Influent** | **Effluent** |  |  |
| Antibiotics | Clarithromycin | Wastewater | China | 2.57-42.1 ng/L | 1.76-5.4 ng/L | Combined treatment of ozonation, UV, and chlorination | Zhang et al., 2021 |
|  | Clarithromycin | Municipal wastewater | Columbia | 40 ng/L | 16 ng/L | Full scale constructed wetlands treatment system | Bayati et al., 2021 |
|  | Amoxicillin | Domestic sewage | India | 172.6 ng/L | 62.5 ng/L | Pre-treatment process for the separation of floating matter and grit, and extended aeration tank for biological treatment | Mutiyar and Mittal, 2013 |
|  | Ampicillin | Wastewater | India | 104.2 μg/L | 12.68 μg/L | Activated sludge treatment process | Mutiyar and Mittal, 2014 |
|  | Trimethoprim | Wastewater | USA | 7 μg/L | 0.21 μg/L | Extended aeration ferrous chloride and sand filtration | Batt et al., 2007 |
|  | Ciprofloxacin | Wastewater | USA | 1.4 μg/L | 0.34 μg/L | Rotating biological contactors and sand filtration seasonal ultraviolet radiation | Batt et al., 2007 |
|  | Tetracycline | Wastewater | USA | 1.1 μg/L | 0.16 μg/L | Activated sludge, nitrification, sand filtration, and seasonal chlorination | Batt et al., 2007 |
|  | Tetracycline | Soil samples | Germany | 86.2 μgkg^−1^ in the 0–10 cm layer;  198.7 μgkg−1 in the 10–20 cm layer;  171.7 μgkg−1 in the 20–30 cm layer; and  No detection at 30 -90cm layer | | No treatment was applied | Hamscher et al., 2002 |
|  | Chlortetracycline | Soil samples | Germany | 4.6–7.3 μgkg−1 in the three sublayers of the soil | | No treatment was applied | Hamscher et al., 2002 |
|  | Sulfamethoxazole | Wastewater | Korea | 7.8 ng/L | 2.1 ng/L | Combined coagulation-sedimentation, filtration (sand), and disinfection treatments | Nam et al., 2014 |
|  | Sulfamethoxazole | Raw domestic sewage | China | 77.6 - 106 ng/L | 11.2 - 19.5 ng/L | Tidal flow constructed wetlands (substrate adsorption, plant uptake, and microbial degradation) | Cheng et al., 2021 |
|  | Sulfamethoxazole | Municipal wastewater | Columbia | 221 ng/L | 85 ng/L | Full scale constructed wetlands treatment system | Bayati et al., 2021 |
|  | Sulfamethoxazole | Wastewater | South Africa | 34.5 μg/L | Less than method limit of detection | Activated sludge process | Matongo et al., 2015 |
|  | Sulfamethoxazole | Biosolids from treatment plant | South Africa | 55.6 ng/g | Less than method limit of detection | Activated sludge process | Matongo et al., 2015 |
|  | Sulfamethoxazole | Urban wastewater | China | 12.9 ng/L | 2.2 ng/L | Anaerobic/anoxic/oxic process and flocculation-sedimentation | Lin et al., 2020 |
|  | Lincomycin | Municipal wastewater | Columbia | 19 ng/L | 9 ng/L | Activated sludge plant with full scale constructed wetlands treatment system | Bayati et al., 2021 |
|  | Azithromycin | Municipal wastewater | Columbia | 709 ng/L | 23 ng/L | Activated sludge plant with full scale constructed wetlands treatment system | Bayati et al., 2021 |
|  | Oxytetracycline | Urban wastewater | China | 287.5 ng/L | 194.9 ng/L | Anaerobic/anoxic/oxic process and flocculation-sedimentation | Lin et al., 2020 |
|  | Triclocarban | Municipal | Columbia | 52 ng/L | 49 ng/L | Full scale constructed wetlands treatment system | Bayati et al., 2021 |
|  | Florfenicol | Urban wastewater | China | 216.6 ng/L | 112.8 ng/L | Anaerobic/anoxic/oxic process and flocculation-sedimentation | Lin et al., 2020 |
|  | Ritonavir | Wastewater | Columbia | 8 ng/L | 5 ng/L | Activated sludge plant with full scale constructed wetlands treatment system | Bayati et al., 2021 |
|  | Miconazole | Wastewater | Columbia | 13 ng/L | 11 ng/L | Activated sludge plant with full scale constructed wetlands treatment system | Bayati et al., 2021 |
|  | Thiabendazole | Municipal wastewater | Columbia | 5 ng/L | 4 ng/L | Activated sludge plant with full scale constructed wetlands treatment system | Bayati et al., 2021 |
|  | Enrofloxacin | Urban wastewater | China | 92.2 ng/L | 52.6 ng/L | Anaerobic/anoxic/oxic process and flocculation-sedimentation | Lin et al., 2020 |
|  | Trimethoprim | Municipal wastewater | Columbia | 392 ng/L | 124 ng/L | Activated sludge plant with full scale constructed wetlands treatment system | Bayati et al., 2021 |
| Analgesics and nonsteroidal anti- inflammatory drugs | Ibuprofen | Wastewater | China | 50-200 ng/L | 37 ng/L | Combined treatment of ozonation, UV, and chlorination | Zhang et al., 2021 |
|  | Ibuprofen | Dry sewage sludge | Spain | 85.9 ng/g | 13.1 ng/g | Solid-phase treatment with fungus *Trametes versicolor* after anaerobic digestion and thermal dehydration from wastewater treatment plant | Rodriguez-Rodriguez et al., 2011 |
|  | Ibuprofen | Wastewater | Korea | 19.6 ng/L | 4.3 ng/L | Combined coagulation-sedimentation, filtration (sand), and disinfection treatments | Nam et al., 2014 |
|  | Ibuprofen | Municipal wastewater | Columbia | 82 ng/L | 74 ng/L | Activated sludge plant with full scale constructed wetlands treatment system | Bayati et al., 2021 |
|  | Ibuprofen | Wastewater | South Africa | 62.82 μg/L | 58.71 μg/L | Activated sludge process | Matongo et al., 2015 |
|  | Ibuprofen | Biosolids | Korea | 48.24 ng/g | 13.15 ng/g | Combined coagulation-sedimentation, filtration (sand), and disinfection treatments | Nam et al., 2014 |
|  | Codeine | Hospital wastewater | Italy | 0.11 μg/L | 0.066 μg/L | Screen and grit removal, conventional activated sludge system including denitrification and nitrification, and secondary sedimentation | Verlicchi et al., 2012 |
|  | Diclofenac | Dry sewage sludge | Spain | 60.3 ng/g | 13.6 ng/g | Solid-phase treatment with fungus *Trametes versicolor* after anaerobic digestion and thermal dehydration from wastewater treatment plant | Rodriguez-Rodriguez et al., 2011 |
|  | Diclofenac | Hospital wastewater | Italy | 0.44 μg/L | 0.28 μg/L | Screen and grit removal, conventional activated sludge system including denitrification and nitrification, and secondary sedimentation | Verlicchi et al., 2012 |
|  | Diclofenac | Wastewater | Korea | 7.8 ng/L | Less than method limit of detection | Combined coagulation-sedimentation, filtration (sand), and disinfection treatments | Nam et al., 2014 |
|  | Acetaminophen | Wastewater | Korea | 106.1 ng/L | 5.0 ng/L | Combined coagulation-sedimentation, filtration (sand), and disinfection treatments | Nam et al., 2014 |
|  | Acetaminophen | Biosolids | Korea | 12.08 ng/g | 7.02 ng/g | Combined coagulation-sedimentation, filtration (sand), and disinfection treatments | Nam et al., 2014 |
|  | Ketoprofen | Municipal wastewater | Columbia | 516 ng/L | 417 ng/L | Activated sludge plant with full scale constructed wetlands treatment system | Bayati et al., 2021 |
|  | Ketoprofen | Municipal wastewater | Korea | 516 ng/L | 417 ng/L | Combined coagulation-sedimentation, filtration (sand), and disinfection treatments | Nam et al., 2014 |
|  | Paracetamol | Municipal wastewater | Columbia | 121 ng/L | 86 ng/L | Activated sludge plant with full scale constructed wetlands treatment system | Bayati et al., 2021 |
|  | Phenazone | Municipal wastewater | Columbia | 28 ng/L | 10 ng/L | Activated sludge plant with full scale constructed wetlands treatment system | Bayati et al., 2021 |
|  | Indoprofen | Municipal wastewater | Korea | 2 ng/L | 1 ng/L | Combined coagulation-sedimentation, filtration (sand), and disinfection treatments | Nam et al., 2014 |
|  | Salicylic acid | Municipal wastewater | Columbia | 106 ng/L | 56 ng/L | Activated sludge plant with full scale constructed wetlands treatment system | Bayati et al., 2021 |
|  | Tolfenamic acid | Municipal wastewater | Columbia | 46 ng/L | 6 ng/L | Activated sludge plant with full scale constructed wetlands treatment system | Bayati et al., 2021 |
|  | Mefenamic acid | Dry sewage sludge | Spain  China | 17.9 ng/g | 3.1 ng/g | Solid-phase treatment with fungus *Trametes versicolor* after anaerobic digestion and thermal dehydration from wastewater treatment plant | Rodriguez-Rodriguez et al., 2011 |
|  | Indomethacin | Hospital wastewater | Italy | 0.16 μg/L | 0.10 μg/L | Screen and grit removal, conventional activated sludge system including denitrification and nitrification, and secondary sedimentation | Verlicchi et al., 2012 |
| Insect repellent | DEET | Municipal wastewater | Columbia | 84 ng/L | 79 ng/L | Activated sludge plant with full scale constructed wetlands treatment system | Bayati et al., 2021 |
|  | DEET | Wastewater | Spain | 50-200 ng/L | 16.7 ng/L | Solid-phase treatment with fungus *Trametes versicolor* after anaerobic digestion and thermal dehydration from wastewater treatment plant | Rodriguez-Rodriguez et al., 2011 |
|  | DEET | Urban wastewater | New Zealand | 1925 ng/L | 94 ng/L | Primary treatment unit and parallel secondary treatment trains of five-stage consisting of anaerobic, anoxic, aerobic, anoxic, and aerobic zones for enhanced nutrient removal with a membrane bioreactor | Kumar et al., 2019 |
| Lipid regulators and statins | Bezafibrate, clofibric acid | Wastewater | China | 2.57-42.1 ng/L | 1.76-5.4 ng/L | Combined treatment of ozonation, UV, and chlorination | Zhang et al., 2021 |
|  | Atorvastatin | Dry sewage sludge | China | 37.8 ng/g | 4.7 ng/g | Combined treatment of ozonation, UV, and chlorination | Zhang et al., 2021 |
|  | Pravastatin | Hospital wastewater | Italy | 0.11 μg/L | 0.54 μg/L | Screen and grit removal, conventional activated sludge system including denitrification and nitrification, and secondary sedimentation | Verlicchi et al., 2012 |
|  | Gemfibrozil | Hospital wastewater | Italy | 0.2 μg/L | 0.11 μg/L | Screen and grit removal, conventional activated sludge system including denitrification and nitrification, and secondary sedimentation | Verlicchi et al., 2012 |
|  | Mevastatin | Hospital wastewater | Italy | 0.17 μg/L | 0.083 μg/L | Screen and grit removal, conventional activated sludge system including denitrification and nitrification, and secondary sedimentation | Verlicchi et al., 2012 |
|  | Pravastatin | Wastewater | Columbia | 917 ng/L | 648 ng/L | Activated sludge plant with full scale constructed wetlands treatment system | Bayati et al., 2021 |
|  | Furosemide | Municipal wastewater | Columbia | 289 ng/L | 164 ng/L | Activated sludge plant with full scale constructed wetlands treatment system | Bayati et al., 2021 |
| Antiretroviral | Ritonavir | Municipal wastewater | Columbia | 289 ng/L | 164 ng/L | Activated sludge plant with full scale constructed wetlands treatment system | Bayati et al., 2021 |
| ꞵ-blockers | Atenolol | Municipal wastewater | Columbia | 575 ng/L | 141 ng/L | Activated sludge plant with full scale constructed wetlands treatment system | Bayati et al., 2021 |
|  | Atenolol | Hospital wastewater | Italy | 2.1 μg/L | 0.073 μg/L | Screen and grit removal, conventional activated sludge system including denitrification and nitrification, and secondary sedimentation | Verlicchi et al., 2012 |
|  | Atenolol | Urban wastewater | New Zealand | 763 ng/L | 237 ng/L | Primary treatment unit and parallel secondary treatment trains of five-stage consisting of anaerobic, anoxic, aerobic, anoxic, and aerobic zones for enhanced nutrient removal with a membrane bioreactor | Kumar et al., 2019 |
|  | Metoprolol | Wastewater | Korea | 37.4 ng/L | 35.1 ng/L | Combined coagulation-sedimentation, filtration (sand), and disinfection treatments | Nam et al., 2014 |
|  | Metoprolol | Municipal wastewater | Columbia | 736 ng/L | 475 ng/L | Activated sludge plant with full scale constructed wetlands treatment system | Bayati et al., 2021 |
|  | Metoprolol | Hospital wastewater | Italy | 0.26 μg/L | 0.18 μg/L | Screen and grit removal, conventional activated sludge system including denitrification and nitrification, and secondary sedimentation | Verlicchi et al., 2012 |
|  | Metoprolol | Urban wastewater | New Zealand | 5244 ng/L | 3097 ng/L | Primary treatment unit and parallel secondary treatment trains of five-stage consisting of anaerobic, anoxic, aerobic, anoxic, and aerobic zones for enhanced nutrient removal with a membrane bioreactor | Kumar et al., 2019 |
|  | Propranolol | Municipal wastewater | Columbia | 91 ng/L | 45 ng/L | Activated sludge plant with full scale constructed wetlands treatment system | Bayati et al., 2021 |
|  | Propranolol | Hospital wastewater | Italy | 0.026 μg/L | 0.018 μg/L | Screen and grit removal, conventional activated sludge system including denitrification and nitrification, and secondary sedimentation | Verlicchi et al., 2012 |
|  | Sotalol | Hospital wastewater | Italy | 0.53 μg/L | 0.32 μg/L | Screen and grit removal, conventional activated sludge system including denitrification and nitrification, and secondary sedimentation | Verlicchi et al., 2012 |
| Anticoagulant | Warfarin | Municipal | Columbia | 6 ng/L | 4 ng/L | Activated sludge plant with full scale constructed wetlands treatment system | Bayati et al., 2021 |
| Antidepressant/antiepileptic | Carbamazepine | Dry sewage sludge | Spain | 25.6 ng/g | 9.1 ng/g | Solid-phase treatment with fungus *Trametes versicolor* after anaerobic digestion and thermal dehydration from wastewater treatment plant | Rodriguez-Rodriguez et al., 2011 |
|  | Carbamazepine | Wastewater | Korea | 10.3 ng/L | 1.6 ng/L | Combined coagulation-sedimentation, filtration (sand), and disinfection treatments | Nam et al., 2014 |
|  | Carbamazepine | Wastewater | South Africa | 2.21 μg/L | 0.91 μg/L | Activated sludge process | Matongo et al., 2015 |
|  | Carbamazepine | Biosolids | Spain | 4.45 ng/g | 1.52 ng/g | Solid-phase treatment with fungus *Trametes versicolor* after anaerobic digestion and thermal dehydration from wastewater treatment plant | Rodriguez-Rodriguez et al., 2011 |
|  | Phenytoin | Municipal | Columbia | 5939 ng/L | 5294 ng/L | Activated sludge plant with full scale constructed wetlands treatment system | Bayati et al., 2021 |
|  | Diazepam | Dry sewage sludge | Spain | 19.3 ng/g | 6.8 ng/g | Solid-phase treatment with fungus *Trametes versicolor* after anaerobic digestion and thermal dehydration from wastewater treatment plant | Rodriguez-Rodriguez et al., 2011 |
|  | Fluoxetine | Municipal | Columbia | 39 ng/L | 7 ng/L | Activated sludge plant with full scale constructed wetlands treatment system | Bayati et al., 2021 |
|  | Fluoxetine | Hospital wastewater | Italy | 0.11 μg/L | 0.044 μg/L | Screen and grit removal, conventional activated sludge system including denitrification and nitrification, and secondary sedimentation | Verlicchi et al., 2012 |
|  | Sertraline | Municipal | Columbia | 46 ng/L | 6 ng/L | Activated sludge plant with full scale constructed wetlands treatment system | Bayati et al., 2021 |
|  | Oxazepam | Municipal | Columbia | 23 ng/L | 16 ng/L | Activated sludge plant with full scale constructed wetlands treatment system | Bayati et al., 2021 |
|  | Paroxetine | Hospital wastewater | Italy | 0.041 μg/L | 0.013 μg/L | Screen and grit removal, conventional activated sludge system including denitrification and nitrification, and secondary sedimentation | Verlicchi et al., 2012 |
|  | Lorazepam | Hospital wastewater | Italy | 0.22 μg/L | 0.12 μg/L | Screen and grit removal, conventional activated sludge system including denitrification and nitrification, and secondary sedimentation | Verlicchi et al., 2012 |
| Antiseptic/disinfectant | Triclosan | Urban wastewater | China | 418 ng/L | 204 ng/L | Anaerobic/anoxic/oxic process and flocculation-sedimentation | Lin et al., 2020 |
|  | Triclosan | Municipal wastewater | Columbia | 232 ng/L | 64 ng/L | Activated sludge plant with full scale constructed wetlands treatment system | Bayati et al., 2021 |
|  | Triclosan | Urban wastewater | New Zealand | 103 ng/L | 23 ng/L | Primary treatment unit and parallel secondary treatment trains of five-stage consisting of anaerobic, anoxic, aerobic, anoxic, and aerobic zones for enhanced nutrient removal with a membrane bioreactor | Kumar et al., 2019 |
|  | Triclosan | Wastewater | Greece | 681.2 ng/L | 139.2 ng/L | Conventional activated sludge secondary treatment and nitrogen and phosphate removal | Kosma et al., 2014 |
| Stimulants/psychoactive | Caffeine | Wastewater | Korea | 36.1 ng/L | 5.8 ng/L | Combined coagulation-sedimentation, filtration (sand), and disinfection treatments | Nam et al., 2014 |
|  | Caffeine | Wastewater | South Africa | 4.48 μg/L | 0.61 μg/L | Activated sludge process | Matongo et al., 2015 |
|  | Caffeine | Wastewater | Greece | 14928.7 ng/L | 189.9 ng/L | Conventional activated sludge secondary treatment and nitrogen and phosphate removal | Kosma et al., 2014 |

**Table S7.** Selected treatment techniques of pharmaceutical and personal care products (PPCPs) indicating the effluent toxicity status.

| **Sample** | **Targeted PPCPs (compounds)** | **Treatment and bioremediation methods** | **Concentration** | **Removal rate** | **Operating conditions** | **Toxicity risk** | **References** |
| --- | --- | --- | --- | --- | --- | --- | --- |
| Influent of industrial wastewater and domestic sewage, wastewater treatment plants effluent, and surface water | Mefenamic acid, trimethoprim, and gemfibrozil | Biological treatment, reverse osmosis | 15 - 3,684 (ng/L) | 40 – 58% | Hydraulic retention time 12.5 h, solid retention time of 12 days | Medium risk to aquatic environment | Dai et al., 2014 |
| Wastewater | Ibuprofen, paracetamol, and clarithromycin | Horizontal flow constructed wetlands | 24 – 23,571 (ng/L) | 54-91% | Hydraulic retention time of 9 to 36 h | High risk | Vymazal et al., 2017 |
| Surface water and wastewater (influent and effluent) | Benzophenone-4 | Tricking filter beds and activated sludge in wastewater treatment plants | 818 – 13,248 (μg/L) | < 15% | Sampling regime air temperature 15 – 20 °C | High risk | Kasprzyk-Hordern et al., 2009 |
| Surface water and wastewater (influent and effluent) | Parabens (preservatives), amphetamine | Tricking filter beds and activated sludge in wastewater treatment plants | 0 - 12,020 (ng/L) | > 90% | Sampling regime air temperature 15 – 20 °C | Low risk | Kasprzyk-Hordern et al., 2009 |
| Dry sewage sludge from wastewater treatment plant | Phenazone, atenolol clarithromycin, bezafibrate, fenofibrate, cimetidine, clarithromycin, and sulfamethazine | Solid-phase treatment with fungus *Trametes versicolor* | 0.5 – 21 (ng/g) | 100% | 42 days incubation at 25 °C. Sample sterilized using autoclaving at 121 °C for 30 minutes | Low risk | Rodriguez-Rodriguez et al., 2011 |
| Wastewater from livestock | Enrofloxacin and tetracycline | Vertical subsurface flow wetlands | 0.7 – 6.1 (μg/L) | 94% and 98% | Environmental temperature of 16 ± 2 °C to 28 ± 8 °C, light exposure | Low risk | Carvalho et al., 2013 |
| Raw domestic sewage | Sulfamethoxazole | Tidal flow constructed wetlands (substrate adsorption, plant uptake, and microbial degradation) | 12.5 – 77.6 (ng/L) | 74 – 85% | Temperature, pH, and dissolved oxygen were kept at 15.8–16.9 °C, 6.84–7.27 and 0.51–0.62 mg/L, respectively | Medium risk | Cheng et al., 2021 |
|  | 4-androstene-3,17-dione |  | 0.54 – 25.7 (ng/L) | 96 – 98% |  | Minimal to low risk |  |
|  | Androsta-1,4-diene-3,17-dione |  | 1.66 – 40.1 (ng/L) | 88 – 100% |  | Minimal to low risk |  |
|  | Climbazole |  | 11.6 – 637 (ng/L) | 86 - 95% |  | Low to medium risk |  |
| Municipal wastewater | Paracetamol, metoprolol, naproxen, trimethoprim, ibuprofen, salicylic acid, ketoprofen, carbamazepine, tolfenamic acid, atenolol, indomethacin, and lincomycin | Activated sludge plant with full scale constructed wetlands treatment system | 9 – 736 (ng/L) | 4 – 87% | Hydraulic retention time of 3.7 days and surface area of 53 ha with four units | Low risk | Bayati et al., 2021 |
| Municipal wastewater | Fluoxetine, azithromycin, propranolol, clarithromycin, and pravastatin | Activated sludge plant with full scale constructed wetlands treatment system | 16 – 917 (ng/L) | 82, 97, 51, 60, and 29%, respectively. | Hydraulic retention time of 3.7 days and surface area of 53 ha with four units | Medium risk | Bayati et al., 2021 |
| Municipal wastewater | Sulfamethoxazole | Activated sludge plant with full scale constructed wetlands treatment system | 85 – 221 (ng/L) | 62% | Hydraulic retention time of 3.7 days and surface area of 53 ha with four units | High risk | Bayati et al., 2021 |
| Urban wastewater | Sulfamethoxazole, oxytetracycline, triclosan | Anaerobic/anoxic/toxic process and flocculation-sedimentation | 1.3 – 718.0 (ng/L) | 21 – 89% | 10% polyaluminum chloride for micro-flocculation and 128 UV-C lamps, 320 W for each lamp for UV disinfection | High ecology risk | Lin et al., 2020 |
| Wastewater | Sulfamethoxazole, clarithromycin, erythromycin | Anaerobic/anoxic/oxic process combined with a moving-bed biofilm reactor for secondary treatment and ultrafiltration, ozonation and ClO_2_ disinfection for tertiary treatments | 1.8 – 3,930.8 (ng/L) | >80% | Flow rate: 4.3 to 200 x 104 m^3^/day, hydraulic retention time: 5 to 21 h, sludge retention time: 13 to 25 days, Temperature: 19.2 to 24.2 °C | High risk | Ben et al., 2018 |
| Wastewater | Ofloxacin, ciprofloxacin |  | 0.6 – 2338.3 (ng/L) | 76 – 87% |  |  |  |
|  | Tetracycline, oxytetracycline |  | 0.4 – 626.9 (ng/L) | >80% |  | Medium |  |
| Excess sludge | Ofloxacin, ciprofloxacin |  | 2.5 – 8,800.5 (ng/L) | 76 – 87% |  |  |  |
| Wastewater | Ciprofloxacin,  norfloxacin | Nitrification, and partially denitrifying with post-filtration process | 0 - 435 (ng/L) | 79 - 87% | Retention time of 12 to 20 h and operative sludge ages between 7 and 16 days | Low | Golet et al., 2002 |
| Urban wastewater | Triclosan and diclofenac | Conventional activated sludge secondary treatment and nitrogen and phosphate removal | 0 – 1,742.5 (ng/L) | 67% | Sludge retention time of 20 days and hydraulic retention time of 22 h | High | Kosma et al., 2014 |
|  | Trimethoprim |  | 0 – 180.3 (ng/L) | 23-91% |  |  |  |
|  | Sulfamethoxazole |  | 0 – 2,626.3 | 58-99% |  |  |  |
